# The behaviour of phenothiazines as catholytes in aqueous-organic redox flow batteries

**DOI:** 10.1039/d5eb00223k

**Published:** 2026-06-17

**Authors:** Nadia L. Farag, Kieran Mylrea, Dominic Hey, Kawarpal Singh, Dominic S. Wright, Clare P. Grey

**Affiliations:** a Yusuf Hamied Department of Chemistry University of Cambridge Lensfield Rd Cambridge CB2 1EW UK cpg27@cam.ac.uk

## Abstract

A series of commercially available phenothiazine dyes was explored for their application in aqueous organic redox flow batteries. Of the dyes explored, Azure-A was found to have the most promising cycling performance and highest solubility, which was improved further with the use of nicotinamide as a ‘hydrotrope’ additive, which improved both solubility and cycle life. However, it was found that only ∼50% of theoretical capacity expected for Azure-A redox could be reached, regardless of cycling conditions. Through *in situ* and *ex situ* NMR, UV/vis and EPR spectroscopy as well as battery cycling using low-concentrations of the dye, this was ascribed to dimerisation of the redox-active species, which takes place at concentrations greater than 10 mM. Because of this, Azure-A is, in effect, only capable of a net 1e^−^ redox process under practical conditions. By combining novel electrochemical impedance spectroscopy processing methods (including distribution of relaxation times and general phase element analysis) with symmetric cell cycling, a degradation mechanism involving polymerisation and passivation of the electrode is proposed as one source of the decrease in capacity with cycling.

## Abstract 2

A series of commercially available phenothiazine dyes was explored for their application in aqueous organic redox flow batteries.

## Graphical abstract

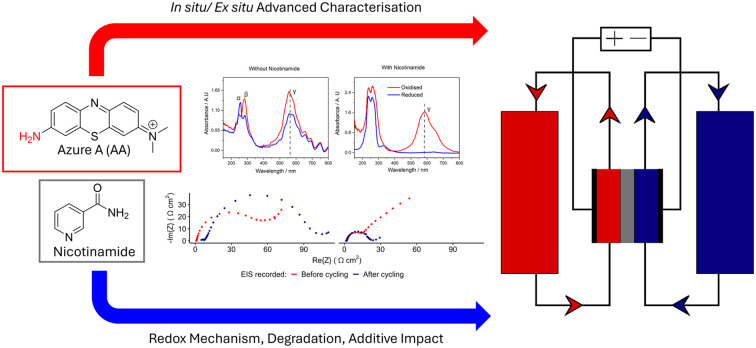

**Broader context**

Aqueous organic redox flow batteries (AORFBs) are a promising technology as an energy storage solution for grid-level storage. However, issues such as poor solubility and low stability plague development of organic aqueous electrolytes, particularly the catholyte side. Phenothiazines have been proposed as a promising family of compounds for use as catholytes in organic RFBs, but only in recent years has their performance in aqueous conditions begun to be evaluated. This paper (to the best of our knowledge) represents the first exploration of the commercially available, non-hazardous, Azure-A as a catholyte in AORFBs. This work is one of few examples using in-depth impedance analysis techniques to understand the degradation mechanism. Overall, our work lays out a workflow/methodology which should be of value to a broad range of researchers in the AORFB area. Finally, while nicotinamide has been previously explored as an additive in AORFBs, our work explores the promising effect on solubility as well as electrochemical performance. The dramatic improvement to cycling performance observed in the presence of NA, apparently resulting from suppression of oligomeric intermediates, is an exciting result not only for applications of AA and other phenothiazines but other families of organic compounds serving as electrolytes in AORFBs.

## Introduction

Redox flow batteries (RFBs) are an exciting emerging technology with the potential to reduce the use of fossil fuels and thus to help mitigate the effects on the climate. The ability to decouple energy and power in RFBs means they are uniquely suited to large grid-scale energy storage and distribution.^1^ RFBs consist of two electrolyte tanks comprising redox-active compounds (the anolyte and catholyte). Upon charging, the catholyte is oxidised and the anolyte reduced, the reverse occurring during discharge. The most well-developed, vanadium-based RFBs have already been implemented commercially in conjunction with renewable energy sources.^2^ In these systems the V^2+/V3+^ redox couple acts as the anolyte and the V^4+/5+^ couple as the catholyte, the main advantage being that crossover of the redox-active species through the membrane is less of an issue as vanadium ions are present on both sides of the cell.^3^ However, due to the high cost, toxicity and limited energy density of the vanadium-based RFB, alternate technologies need to be considered to facilitate RFB use on a wider scale.

Organic redox pairs offer the potential for both reduced cost and toxicity and have been used in both organic RFBs (ORFBs) and aqueous organic RFBs (AORFBs). It must be noted that aqueous-based electrolyte systems suffer from a reduced potential window (∼1.3 V) compared to those using organic solvents due to the reduced redox window of water; the organic redox-active electrolytes generally show poorer solubility and lower stability, in comparison to their inorganic counterparts.^4^ Despite this, the advantages of aqueous electrolytes (including safety, cost, improved kinetics and more), make AORFBs the more attractive system for the future. Aqueous-compatible anolytes have been extensively explored, while comparatively few catholytes have been reported, and those that are reported rarely meet the standards of performance set by their anolyte counterparts. Notable catholytes for AORFBs include TEMPO (2,2,6,6-tetramethylpiperidine-1-oxyl), viologen, ferrocene, ferricyanide and their derivatives.^5–10^ However, high performance and solubility thresholds have yet to be met with a non-toxic redox couple. Based on estimations by the US Department of Energy, the target solubility of organic electrolytes for AORFBs is 2 M for a 1e^−^ redox couple.^4^

**Fig. 1:**
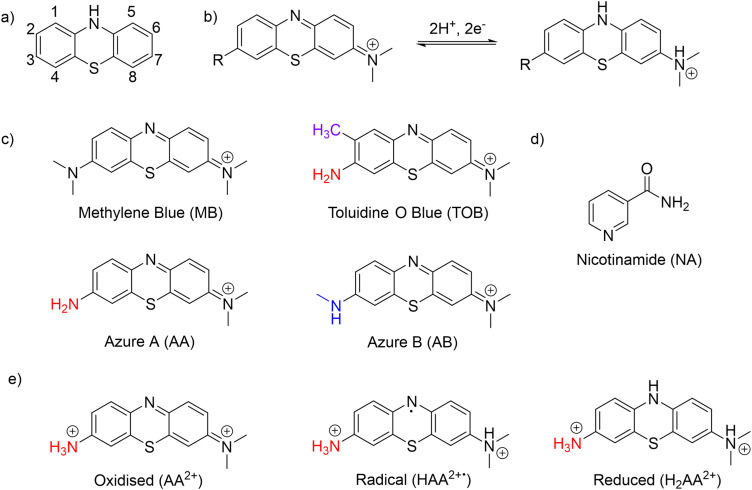
(a) Numbered molecular structure of phenothiazine and (b) the 2e^−^ proton-coupled redox process of a phenothiazine dye. (c) Molecular structures of the phenothiazine dyes methylene blue (MB), toluidine-O blue (TOB), Azure-A (AA) and Azure-B (AB) in their oxidised state; as purchased, Cl^−^ is generally the counter ion. (d) Molecular structure of nicotinamide (NA). (e) AA in the fully-oxidised state and protonated form (AA^2+^), radical state (HAA^2+^˙) and fully-reduced, protonated state (H_2_AA^2+^); these protonated ions are likely to be present in acidic electrolytes.

Phenothiazines (Fig. 1a) have been proposed as electrolyte materials in non-aqueous redox flow batteries, showing promise due to both highly reversible electrochemistry, and ease of synthesis and post synthetic modification.^11^ Despite this, there have been remarkably few explorations in aqueous conditions. Fig. 1b shows the reported redox mechanism common to phenothiazines (a concerted two-electron proton-coupled electron transfer).^12^ The commercial dye methylene blue (MB) (Fig. 1c) was first proposed as a catholyte for RFBs by Kosswattaarchchi *et al.* in 2018. However, they found that MB solubility in 0.5 M H_2_SO_4(aq)_ was extremely limited, with a maximum solubility of only 100 mM.^12^ In 2019 C. Zhang *et al.* found that the poor solubility could be overcome with a mixed acetic acid and 3 M H_2_SO_4(aq)_ electrolyte system, and they were able to achieve remarkable solubility and performance. The maximum solubility was found to be ∼1.8 M and battery cycling with 1.2 M MB showed capacity retention over 160 cycles (capacity fade was reported to be 0.025% per cycle), while at 1.5 M the electrolyte showed a slightly increased capacity fade over 50 cycles (0.074% per cycle).^13^ This work demonstrated the performance heights that can be reached by phenothiazine derivatives and highlighted the importance of the chosen electrolyte. In 2023 this same system was explored by Y. Zhang *et al.* using *in situ* nuclear magnetic resonance (NMR) and *ex situ* electron paramagnetic resonance (EPR) spectroscopy, further elucidating the redox mechanism and providing insight into a radical intermediate formed during cycling.^14^

More recently, M. Zhang *et al.* explored how functional group positions and electronic properties impact the redox activity of phenothiazines, finding that electron-donating groups in the 3- and 7- positions (Fig. 1a) resulted in reversible redox activity in aqueous conditions. Furthermore, a phenothiazine with 2-hydroxyethyl methyl amino groups was synthesised, having reversible redox activity and solubility up to 1.3 M in 3 M H_2_SO_4_.^15^

When considering the development of aqueous electrolytes, solubility and electrolyte environment are key to achieving optimum performance. Organic compounds that exhibit high solubility in basic conditions are likely to have low-to-zero solubility in acidic conditions and *vice versa*. While it is known that pH has a significant effect on electrochemistry and redox potential when there is a proton-coupled electron transfer, the influence of pH remains underexplored and is rarely taken advantage of in RFB systems.^16^ As well as pH control, additives can be used to improve solubility.^17^ Nicotinamide (NA, Fig. 1d), a so-called ‘hydrotope’ (an amphiphile that solubilises hydrophobic molecules in aqueous media), has been used in AORFBs to enhance aqueous solubility of organic compounds at a variety of pHs.^18^ In addition to applications in AORFBs, NA is extensively used in the pharmaceutical industry to enhance water solubility.^19^ Whilst the exact mechanism of the solubilisation is not completely understood, it is generally accepted that hydrogen bonding and π-stacking (or NA aggregation) interactions dominate.^20,21^

While the performance of MB in AORFBs is good, it is toxic and an irritant, as such there needs to be further work done into non-hazardous phenothiazines.^22^ Therefore commercial phenothiazine dyes similar to MB, namely toluidine-O blue (TOB), Azure-B (AB) and Azure-A (AA), (Fig. 1c) were chosen for the current study due to their low cost, availability of materials and to compare the effects of methylation at the ring or N-atoms on the solubility and electrochemical performance of the MB molecule. TOB and AA reportedly have no known hazards (at least as received) and are thus ideal for large-scale applications. When dissolved in the 1 M H_2_SO_4_ electrolyte used in this work, the dyes are further protonated, as shown in Fig. 1e for AA.

AA in combination with NA was first identified in this work *via* electrochemical studies as the most promising candidate for AORFBs due to its high solubility and stable cycling performance. AA was therefore studied in the most detail, with and without NA, by using a combination of *in situ* and *ex situ* NMR, EPR and UV-vis methodologies, and impedance studies, to understand the redox mechanism, observed cycling performance and degradation mechanisms.

## Experimental

Chemicals used were purchased from Sigma Aldrich, ChemCruz, MP Biomedicals, Thermo Scientific, Alfa Aesar and Fischer Chemical. All chemicals were used as supplied, unless otherwise specified in the text. All phenothiazines were bought as cations with a chloride counter ion and specified to have a minimum dye content of between 70 and 80%.

### Electrochemistry

All electrolyte solutions were prepared under inert atmosphere, using dried compounds and de-gassed solvents to prevent oxygen in the system inferring with electrochemical measurements. Where 1 M H_2_SO_4_ has been used as the electrolyte pH has been assumed to be 0.

A 200-VSP potentiostat from biologic was used for all long-term electrochemical measurements. CV and corresponding EIS were measured using a three-electrode cell consisting of a glassy carbon working electrode (Biologic A-012744, 3 mm), platinum wire counter electrode and a Ag/AgCl reference electrode. All CV measurements were run with a N_2_ overpressure to limit air exposure. All solutions for CV were prepared under inert atmosphere. Electrochemical measurements for *in situ* experiments were taken using a portable biologic SP-150 potentiostat.

All flow cell measurements were conducted under an inert N_2_ atmosphere in a Perspex glove box with a constant flow of N_2_. A commercial flow cell from Scribner Associates was used. The cell was comprised of anodised aluminium with flow in and outputs, gold-plated copper current collectors, graphite etched with a serpentine flow field and 0.7 mm thick Viton gaskets. Each electrode contained three 5 cm^2^ sheets of Sigracet 39 AA carbon paper (FuelCellStore). A torque wrench was used to provide an even 2 Nm seal across the cell (each bolt). Nafion 212 membranes were soaked in deionised water and heated to 80 °C for *ca.* 20 minutes, before soaking in 5% hydrogen peroxide solution for a further 35 minutes and finally stored in 0.1 M H_2_SO_4_ in deionised water. These pre-treated membranes were used as the ion-transport membrane in all cells. Masterflex® L/S® 0775-10 peristaltic pumps, Chem-durance® L/S® Bio tubing, polyether-ether-ketone (PEEK) fittings (Diba, Omni-LokTM, Inv. Cone, and barbed adaptors) and perfluoro-alkoxy-alkane (IDEX, PFA Natural 1/AA″ OD, AA ID) tubing were used for flow and electrolyte transport when cycling. Cycling conditions are described where data is reported.

### EIS, DRT and GPE

EIS data were recorded using potentiostatic electochemical impedance spectroscopy (PEIS), with perturbation amplitude 10 mV and a frequency range of 200 kHz to 0.1 Hz for full cell EIS and 200 kHz to 5 Hz for symmetric cell EIS. Before DRT calculation, high frequency inductive data was removed, resulting in slight differences in the upper frequency cutoff before DRT transformation, leading to variable *τ* limits, regardless all datasets include the full region of interest (with *τ* = 10^−5^ to 10^−3^ s).

DRT transformation was achieved using the pyDRTtools open-source software.^23,24^ The form of the DRT transformation used in this case is:
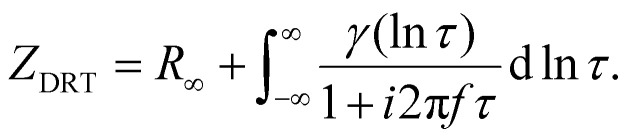


The DRT kernel utilised is non-generalised,^25^ so inductive and diffusive/capacitive low-frequency EIS responses are not well modelled. Inductive contributions are removed by filtering out high frequency data with Im{*Z*} > 0. However, due to the multifaceted nature of the low-frequency EIS response low frequency modelling and subtraction of diffusive and capacitive contributions is not undertaken in the current work – with likely contributions from the flow set-up, ion transport through the porous carbon paper,^26^ the slow vanadium charge transport kinetics^26^ and also diffusion and capacitive effects on a range of different length scales.^27^

General phase element analysis was undertaken using a self-written script in *R*. The expressions for 2D- and 3D-capacitance values used in the current work come from ref. 30 and are:
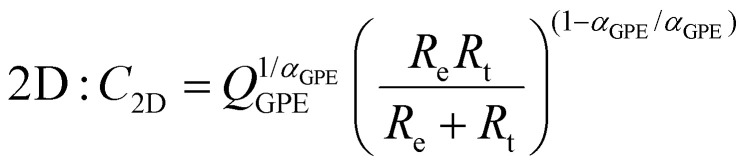

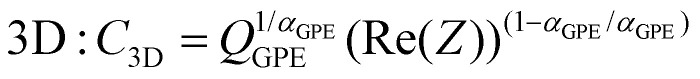
where:
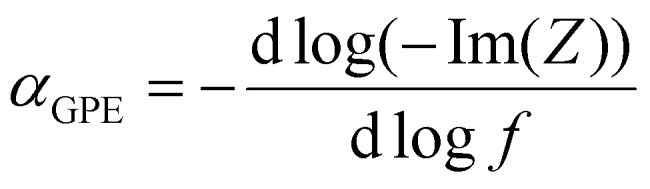

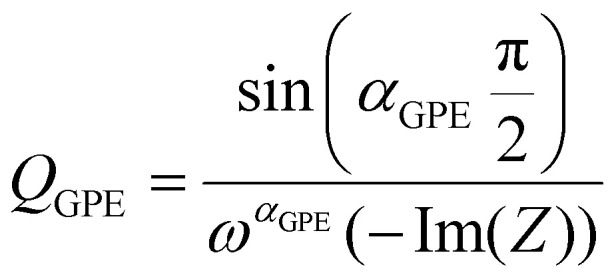
*R*_e_ = resistance associated with electrolyte (in Ω cm^2^), *R*_t_ = charge transfer resistance of a faradaic reaction (in Ω cm^2^), Im(*Z*) = imaginary component of recorded EIS data (in Ω cm^2^), *f* = frequency of recorded EIS (in Hz), *ω* = 2π*f* (in Hz).

### NMR

^1^H and ^13^C NMR spectra were obtained using a 400 MHz Bruker Avance III HD spectrometer outfitted with a BBO smart probe and results analysed using TopSpin, 528PP NMR tubes were used. Each experiment was performed with 16 scans under thermal-equilibrium conditions with 10 s of recycle delay, after optimizing the π/2 pulse length. The acquisition time for each experiment was 3 s. All experiments were, unless otherwise stated, measured at room temperature. *In situ* NMR measurements were performed using the flow set-up previously described with an SP-300, biologic SAS or IVIUM cycler, benchtop EPR (MS5000, Magnettech) and 300 MHz NMR spectrometer (Bruker). Solutions were flowed through reservoirs, bottom to top, in the respective spectrometers. Each time-resolved 1D ^1^H NMR spectrum was obtained in 64 s with 16 scans to achieve desired signal-to-noise ratio. The flow rates were optimized to achieve the signal under thermal equilibrium without losing the signal due to longer-longitudinal relaxation rate *T*_1_.

### UV/vis

*In situ* UV/vis used the same flow cell set-up as described above, solutions were flowed through the cell before being flowed through an optical cuvette (100 μm optical path-length flow cell (Ocean Optics FIA-USP-100)) and back into the catholyte tank. The UV/vis was measured using a deuterium-tungsten light source (Ocean Optics 2000-BAL) connected to the optical flow cell using 25 cm optical fibres (Ocean Optics QP400-025-SR-BX), a mirroring optical fibre and UV/vis spectrophotometer (Ocean Optics Flam-S-UV-Vis). *Ex situ* UV/vis was measured using the same 100 μm optical path length flow cell for samples >1 mM AA or a 1 cm optical path length quartz cuvette for samples <1 mM AA. All *ex situ* measurements were taken with a Lambda 750 UV/VIS/NIR Spectrometer (PerkinElmer).

### Density functional theory (DFT)

DFT calculations were performed using a computer cluster (Odyssey HPC cluster) with Gaussian 16. Structures were optimised at the B3LYP/TZVP level of theory.^28,29^ CAM-B3LYP was used for time-dependant DFT to predict UV/vis spectra for these optimised structures.^30^ UV/vis spectra were simulated using the formulae described by Frisch *et al.*^31^

## Results and discussion

### Solubility and electrochemical performance

Table 1 presents solubility data determined in the current study for TOB, AB and AA, with and without NA, along with the redox potentials of these dyes in 1 M H_2_SO_4_, with maximum solubilities being measured using the Beer–Lambert rule by UV/vis spectroscopy (detailed explanation in the SI, Fig. S1).^32^ From these data it can be seen that 1 M NA drastically improves the maximum concentrations of TOB, AB and AA compared to those measured with the use of H_2_SO_4_ alone. The high solubility of AA in the presence of NA is particularly noteworthy in that it achieves the 2M solubility DOE target for practical RFB operation, set for a 1e^−^ redox process.^4^ Furthermore, since it is in theory a two-electron (2e^−^) system, it may be possible to double the energy density over that of the target value. The maximum solubility of MB in 1 M H_2_SO_4_ is only 15 mM.^33^ Interestingly, the addition of NA to MB results in the formation of a salt, which precipitates from solution under these conditions (100 mM MB in 1 M H_2_SO_4_ + 1 M NA) suggesting that the NH/NH_2_ groups of the phenothiazine play a crucial role in the interaction and subsequent solubilisation with NA.

**Table 1:** Solubilities and redox potentials (*vs.* SHE) of phenothiazine dyes, TOB, AB and AA

Compound	Solubility in 1 M H_2_SO_4(aq)_	Solubility in 1 M H_2_SO_4(aq)_ + 1 M NA	Redox potential in 1 M H_2_SO_4(aq)_
TOB	3 mM	161 mM	∼0.55 V
AB	98 mM	730 mM	∼0.50 V
AA	915 mM	2 M (2043 mM)	∼0.45 V

Cyclic voltammetry (CV) was used to assess the suitability of the dyes as catholytes in AORFBs, and a summary of the redox potentials in 1 M H_2_SO_4_ is given in Table 1; in all cases 1 mM of dye was used. All three dyes exhibit seemingly reversible redox processes, the voltages of these varying between 0.45–0.55 V *vs.* the standard hydrogen electrode (SHE). The shift towards lower potential moving from TOB to AB to AA is broadly in line with the decreasing electron-donating ability of the functional groups. From the Nernst equation, the peak-to-peak separation should equal 59 mV per *n*, where *n* is the number of electrons in a reversible process.^34^ The recorded separation was ∼40 mV for all of the dyes, suggesting a 2e^−^ redox process. The presence of NA in the supporting electrolyte appeared to produce minimal differences in peak heights, shapes and redox potentials (Fig. S2). However, the oxidative peak is slightly broader, though this difference is small and could be accounted for by minor variations in environmental conditions, such as temperature.

**Fig. 2:**
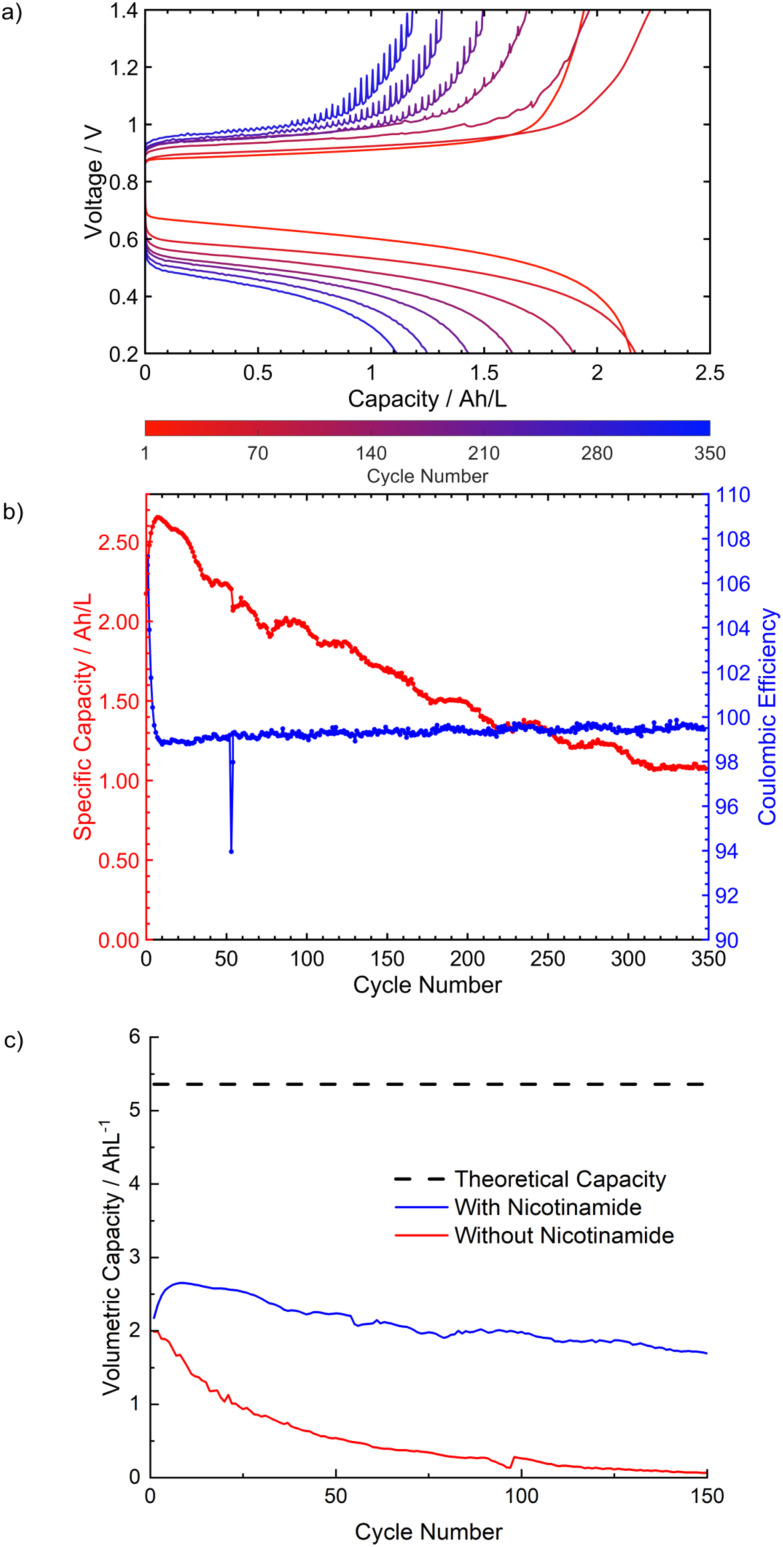
(a) Long-term battery cycling of an AORFB comprising 100 mM AA against 300 mM VCl_2_ and 75 mM VCl_3_ in 1 M H_2_SO_4_ with 1 M NA between 0.2 and 1.4 V with a current of 50 mA (10 mA cm^−1^). The battery was first discharged to 0.2 V before charging to 1.4 V, with every 50 cycles plotted; periodic oscillations in later cycles are tentatively ascribed to pump issues. (b) Capacity (red) and coulombic efficiency (blue) against cycle number for the same cell. (c) Comparison plot of capacity *versus* cycle number for the first 200 cycles of AA with (blue) and without (red) NA; theoretical capacity based on a two-electron redox couple is highlighted by the dotted line.

AB and AA (100 mM solutions) were employed as catholytes in lab-scale AORBFs using either 1 M H_2_SO_4_ or 1 M H_2_SO_4_ + 1 M NA catholyte solutions against VCl_2_ : VCl_3_ in a 4 : 1 molar ratio as the anolyte (300 mM : 75 mM). Due to limited solubility, TOB (100 mM) could only be cycled in 1 M H_2_SO_4_ + 1 M NA (Fig. 2 for AA; AB and TOB cycling data can be found in Fig. S4–S6). In 1 M H_2_SO_4_ there was a rapid capacity fade of *ca.* 1.45% per cycle for AB and AA over the first 50 cycles, whereas with NA these capacity fades were reduced to 1.05% and 0.34% per cycle, respectively. In comparison, 100 mM TOB in 1 M H_2_SO_4_ + 1 M NA had a capacity fade rate of 0.16%. Despite the improved performance of AB with NA, the cycling stability was poor, and whilst TOB exhibited the most stable electrochemical cycling it also had the lowest solubility. Consequently, AA was selected for further cycling studies. As previously stated, all dyes were purchased and deployed in the oxidised state and, due to the strongly acidic nature of the supporting electrolyte, all cycling commenced with the catholytes in the oxidised (and protonated) state (AA^2+^ in the case of AA, Fig. 1e), hence the greater excess of VCl_2_ (V^2+^) used in the anolyte. All cells were discharged first, reducing the catholyte to H_2_AA^2+^, in the case of AA, and oxidising the anolyte to V^3+^, before continuing to cycle.

The theoretical capacities (calculated based on a 2e^−^ process) of all of the catholyte candidates tested at 100 mM were not reached and attempts to optimise cycling conditions resulted in little improvement. Cycling the TOB-containing AORFBs only reached 62% of the theoretical capacity, while AA fared even worse at 49% capacity. Higher concentrations led to increases in capacity for AA: cycling at 200 mM instead of 100 mM roughly doubled the recorded capacity, but this still only reached *ca.* 50% of the theoretical capacity at 200 mM, thus demonstrating that the low capacity is not a result of poor solubility.

To assess the impact on the AA electrochemistry of a potential redox-shuttle mechanism involving vanadium crossover (crossover being confirmed below with EPR spectroscopy), a cell was cycled using a double Nafion 212 membrane (two sheets). While this does appear to reduce the rate of crossover, as evidenced by the reduced capacity-fade rate, no additional capacity was gained above that seen using a single membrane (Fig. S3). Therefore, crossover is not the source of the lower-than-expected capacity for a two-electron couple. This conclusion is further supported by the fact that the rate of capacity fade did not increase with cycle number, as would be expected if crossover was the dominant source of the lower-than-expected capacity. Instead, a near-linear capacity fade is observed here. Therefore, it was necessary to reconsider the redox mechanism and any additional, capacity-limiting processes that occur during cycling.

### Spectroscopic studies of cycling

**Fig. 3:**
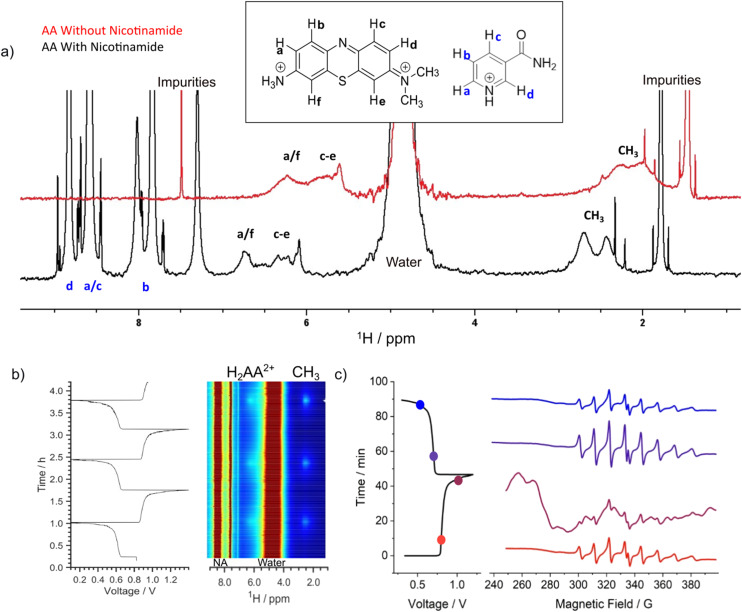
(a) ^1^H NMR (700 MHz) spectra of a saturated solution of AA^2+^ in 1 M H_2_SO_4_ in D_2_O with and without 1 M NA. (b) *In situ*^1^H NMR (300 MHz) spectra of 200 mM AA in 1 M H_2_SO_4_ with 1 M NA in D_2_O cycled between 0.2 and 1.4 V with a current of 50 mA (10 mA cm^−1^) the peak at *ca.* 6.0 ppm represents peaks a–f of H_2_AA^2+^ (labelled H_2_AA^2+^) and the peaks at *ca.* 2.1 ppm are the methyl peaks of H_2_AA^2+^ (labelled CH_3_). (c) *Ex situ* EPR spectra extracted from a battery cycled with the same electrolytes, under the same conditions at the start of the first charge (red), end of charge (dark red), start of the discharge (purple) and end of discharge (blue).

Fig. 3 shows NMR and EPR spectroscopic studies undertaken on AA in 1 M H_2_SO_4_/1 M NA. It was challenging to obtain strong signals for the AA^2+^ peaks using NMR spectroscopy. Even when the NA peaks were relatively sharp, the AA^2+^ peaks remain broad and poorly defined (Fig. 3a). The broadening is not caused by NA as the resonances for AA^2+^ are broad regardless of its presence. This signal broadening could be the result of a small concentration of radicals that interact with the AA^2+^ ions, and EPR of the same samples showing evidence of nitrogen (and possibly HAA^2+^˙) radicals in the solution (Fig. S7). Additionally, aggregation of the AA molecules/ions in solution could broaden peaks as this might restrict their tumbling motions (see below). *In situ*^1^H NMR spectroscopic studies during cycling also confirm the presence of radicals in the AA system. Any AA^2+^ peaks are expected between 6–7 and 2–3 ppm, and when cycling, these peaks are reversibly lost during charging (Fig. 3b). Indeed, only extremely weak AA^2+^ peaks were seen even before the cycling was commenced: it was only when the cell was completely discharged that any signals, presumably from the reduced diamagnetic ion, H_2_AA^2+^, are observed in the ^1^H NMR (see slices of *in situ* NMR spectra in Fig. S8). The peaks are very broad with much lower intensities than expected given the known concentrations or completely absent at most states of charge (SOC), which is presumably at least in part due to the AA ions interacting with paramagnetic intermediates. As only the AA-related peaks are affected it can be concluded that the unpaired electrons are localised on the AA molecules alone and not the NA. A second possible explanation for the almost complete disappearance of the AA peaks upon charging (nominally on going from H_2_AA^2+^ to AA^2+^) is an increased tendency to aggregate in the fully-oxidised form: aggregation would further broaden the peaks, making it appear as though they are disappearing on charge (oxidation). Initial experiments suggest that the line broadening of these spectra is extremely dependent on temperature, providing one explanation for the disappearance of the ^1^H NMR spectra before cycling even commences (Fig. S9).

Interestingly, continued battery cycling for over 50 charge/discharge cycles led to broadening of the water peak and loss of signal intensity across the spectrum; this is most likely to be due to vanadium-radical crossover through the RFB membrane (Fig. S10). Using a larger excess of vanadium anolyte did not increase cell capacity, suggesting that the diminished capacity achieved is not the result of the observed vanadium crossover. It is unclear whether the addition of NA had any effect on crossover, however, it is possible that the interactions between NA and AA increase the effective size of the AA molecules, reducing crossover, though this is unconfirmed here.

To investigate the radicals formed during AA cycling further, and any potential vanadium crossover, *ex situ* EPR spectroscopy measurements were performed on the catholyte solution. Aliquots were taken periodically during cell cycling and their spectra measured (Fig. 3). An EPR signal from the vanadium radical VO^2+^ (V^4+^) (with its characteristic ^51^V hyperfine splitting) was present even on the first charge (red spectrum), indicating that crossover is rapid when using Nafion 212 as the membrane. This cross-over could involve either of the V^2+/V3+^ ions, these ions being rapidly oxidised to VO^2+^ by the catholyte solution/positive electrode. While only the VO^2+^ ion can be seen at or around the discharged state, in the charged state (dark red spectrum, Fig. 3c) there are clearly other radical species present. These are difficult to deconvolute from the dominant VO^2+^ signals, but they may arise from the expected radical species generated by the one-electron reduction of AA; a singlet (superimposed upon a broader more featureless signal) is observed at all states of charge at a magnetic field of approximately 335 G, which aligns well with the observed radical signal present even in a sample of AA in 1 M H_2_SO_4_ (Fig. S11). A singlet is not expected for a radical with electron density potentially delocalised over three nitrogen atoms (see for example the EPR spectrum of the radical formed on reduction of MB^14^), so its origin is not clear. One possible explanation for this signal is that it originates from a dimer formed from a radical bound to the diamagnetic oxidised ion so that exchange of the electron between the two ions washes out the hyperfine interaction. The EPR signal reduces at the top of charge (Fig. 3c, blue trace), but this is largely due to oxidation of VO^2+^ (V^4+^) to VO_2_^+^ (V^5+^), which is expected at 1.0 V *vs.* SHE and so may not reflect any changes to the AA system.^35^

**Fig. 4:**
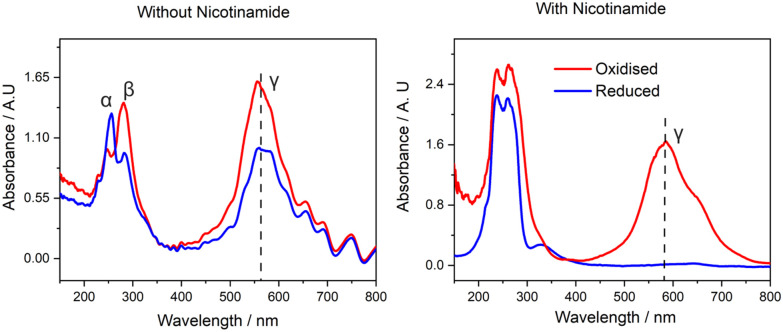
*In situ* UV vis spectra of 10 mM AA in 1 M H_2_SO_4_ without and with 1 M NA measured during cycling. Spectra are shown after the first charge (oxidised) and after the first discharge (reduced).

*In situ* UV/vis spectra were measured for 10 mM AA in both 1 M H_2_SO_4_ and 1 M H_2_SO_4_ + 1 M NA during RFB cell operation (Fig. 4). Bands between 200–300 nm (labelled α and β) correspond to the π–π* transitions within the aromatic frameworks. The α and β bands are obscured by the NA bands in the NA-containing electrolytes, whose spectra also have absorptions between 200–300 nm. However, in the absence of NA the intensity of the α bands alternate with the β band during cycling, with the α band diminishing during charging and the β band growing, the opposite occurring upon discharge. During charging (oxidation of the catholyte) a broad peak at *ca.* 550 nm without NA and *ca.* 575 nm with NA (marked in both cases by a dotted line) also grows in, labelled γ. When discharging, γ decreases in intensity; this effect is more pronounced in the presence of NA as γ is lost completely, while without NA its intensity is only diminished in the discharged state. An example *ex situ* spectrum can be seen in Fig. S12.

In an attempt to identify the species present in the reduced states, density functional theory (DFT) calculations of the monomer were used to predict UV/vis spectra of the fully-oxidised (charged) state (AA^2+^), the radical generated by 1e^−^ reduction of AA (HAA^2+^˙), and the fully-reduced (discharged) state (H_2_AA^2+^) (Fig. S13). In all cases two peaks in the frequency region of the α and β bands were predicted. While the UV/vis spectra of HAA^2+^˙ and H_2_AA^2+^ are qualitatively similar, the intense, broad, bands around 500–600 nm (*γ*) are only seen for the oxidised form, AA^2+^ and can in principle be used to track the SOC of the battery. Furthermore, given that the γ-peak is only predicted for the fully oxidised state, this suggests that fully oxidised AA^2+^ is formed – at least to some extent – during cycling. However, it is important to note that these DFT calculations have only considered the monomer; a fuller analysis requires an exploration of the effect of dimerization of the spectra, as performed in neutral solutions by Gilani *et al.*^36^

#### The role of dimerization

AA has been reported previously by C. Zhang *et al.* to have a 2e^−^ process in 3 M H_2_SO_4_, as outlined in Fig. 1a, and chemically similar MB exhibits 2e^−^ redox behaviour when cycling in an AORFB.^13^ By studying the same MB AORFB system, Y. Zhang *et al.* discovered, *via in situ* NMR and EPR, that while there was a one-step 2e^−^ proton-coupled redox process taking place, there are also radicals present during cycling (most abundantly at 50% state of charge), which they attributed to a comproportionation reaction between the oxidised and reduced species in solution.^14^ Considering the *in situ* NMR spectra reported here, AA resonances were only seen clearly at the end of the discharge, suggesting that a diamagnetic anion – likely containing the fully reduced H_2_AA^2+^: if the radical HAA^2+^˙ was the predominant species, the resonances would likely be washed out. Thus, the combination of the UV/vis, which suggests AA^2+^ is present, and the NMR studies that observe a diamagnetic reduced species, appears to support the 2e^−^ redox mechanism reported in the literature (Fig. 1b). Given this, the low capacity achieved for AA in the RFB studies is surprising (*ca.* 50%).

An alternative explanation for the low capacity is that a diamagnetic dimer ion (*i.e.*, observable by NMR) is present in the fully reduced state. Previous work in the AORFB field has suggested that the dimerization of quinones can significantly reduce accessible capacity.^37^ For example, Carney *et al.* suggested that concentration-dependant quinone dimerisation was the cause of limited capacity in 9,10-anthraquinone-2,7-disulfonic acid (AQDS) systems, where only 50% of the theoretical capacity could be accessed.^37^ This work concluded that AQDS preferentially dimerised at concentrations above 10 mM and this dimerisation limited the energy density of the system as electrons were shared across two molecules instead of one. The second redox couple can no longer be accessed within the relatively limited voltage window of water. With more direct relevance to our study, there have been reports that AA undergoes dimerisation in aqueous media, with this aggregation likely being driven by π-stacking interactions.^36,38^ Interestingly, this effect is not seen in MB systems, since the complete methylation of the NH_2_ groups presumably introduces sufficient steric bulk to prevent dimerisation, so that the theoretical capacity based on a 2e^−^ process is obtained.^13^

**Fig. 5:**
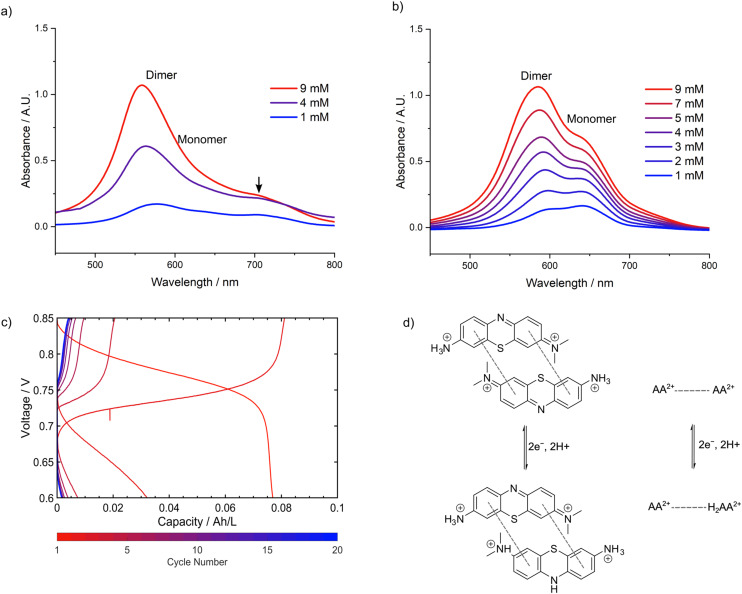
(a) UV/vis of varying concentrations of AA in 1 M H_2_SO_4_ without, (b) and with 1 M NA, (c) AORFB Battery cycling of 2 mM AA against 10 mM VCl_2_ and 5 mM VCl_3_ in 1 M H_2_SO_4_ with 1 M NA, cycled at 1 mA (0.2 mA cm^−1^) first to 0.6 V then to 0.85 V, with every 2 cycles plotted, theoretical capacity is 0.107 Ah L^−1^, (d) proposed AA dimer and redox mechanism.

UV/vis was measured for solutions ranging from 1 to 9 mM in 1 M H_2_SO_4_ without and with NA (Fig. 5a and b), allowing assignment of the peak at *ca.* 575 nm (peak γ), also seen in Fig. 4c and d (for the 10 mM solution) to a dimer, on the basis of its increase in relative intensity with increasing AA concentration. These assignments are in agreement with earlier UV studies performed at neutral pH, which similarly assigned the 575 nm peak to a dimer and furthermore showed that dimerisation dominates at concentrations equal to or greater than 10 mM of AA.^38^ A new peak at an even longer wavelength (*ca.* 650 nm) is now clearly seen in the presence of NA that can be assigned to the monomer, the dimer-to-monomer ratio increasing as the AA concentration increases, consistent with the assignments of the two peaks (and with a monomer-dimer equilibrium). Without NA, a weak, overlapping third peak at >700 nm (indicated by an arrow) is more clearly seen although it is also visible as a shoulder in the spectra obtained with NA. In a previous UV study by da Silva *et al.*, this peak was assigned to a J-type or head-to-head dimer, while the more intense “γ” peak was assigned to a sandwich-type dimer.^38^ The weak monomer peak is also still present. As the AA concentration is increased, the intensity of the sandwich-type dimer grows the fastest, while monomer absorption is much weaker at all concentrations, suggesting a greater tendency to aggregate in the absence of NA. This dimerisation could provide a further explanation for the broadened NMR signals observed for AA^2+^ (discussed previously). However, it should be noted the dimer discussed in the previous study, with its very similar UV signals to those seen here, has been assigned to the dimerisation of the oxidised but deprotonated ion, AA^1+^, rather than AA^2+^. Certainly, dimerisation of the lower-charged species should be more favourable and so the existence of the AA^1+^ dimers must be considered. Future NMR and UV/vis studies will focus on the role of pH on both the dimerisation process, and the effect of protonation of the observed NMR/UV/vis signals.

These results suggest that at high concentrations, the fully reduced species may also be a dimer rather than H_2_AA^2+^. Surprisingly, this species does not seem to be in rapid equilibrium with the radical, as a ^1^H signal is observed by *in situ* NMR spectroscopy for the fully reduced solution, and not the oxidised solution, the latter clearly either containing residual radicals or larger aggregates of AA^2+^, at least at higher concentrations.

Since the equilibrium between AA monomers and dimers would be shifted further towards dimersation at higher concentrations, we next studied the effect of concentration on the capacity of the RFB. Cells containing a low concentration of AA of 2 mM were now able to access around 85% of the theoretical capacity based on the 2e^−^ mechanism (maximum capacity reached is 0.091 Ah L^−1^, theoretical capacity is 0.107 Ah L^−1^, Fig. 5c). However, cycling stability was poor. This increased degradation is tentatively ascribed to reduced dimerisation, likely increasing the concentration of the radicals HAA^2+^˙ and H_2_AA^2+^, and accelerating electrolyte degradation; the degradation mechanism is discussed later in this work.

At high concentrations, we tentatively propose that only one molecule is reduced or oxidised per dimer during discharge and charge (as illustrated in Fig. 5d), with the intermolecular interactions between them preventing the second molecule from being electrochemically reduced within the voltage window studied.

Dimerisation might also suggest a greater propensity for comproportionation to the radical given that we have just proposed that the reduced dimer comprises one fully reduced anion and one fully oxidised ion. The observation of only a weak radical signal seen throughout measurements suggests that the dimer must be significantly lower in energy than the radical dimer. These species could alternatively be viewed as singlet and triplet states of the radical dimer, with the singlet (diamagnetic) system being noticeably more stable.

### Electrochemical impedance investigation of the degradation mechanism of AA

**Fig. 6:**
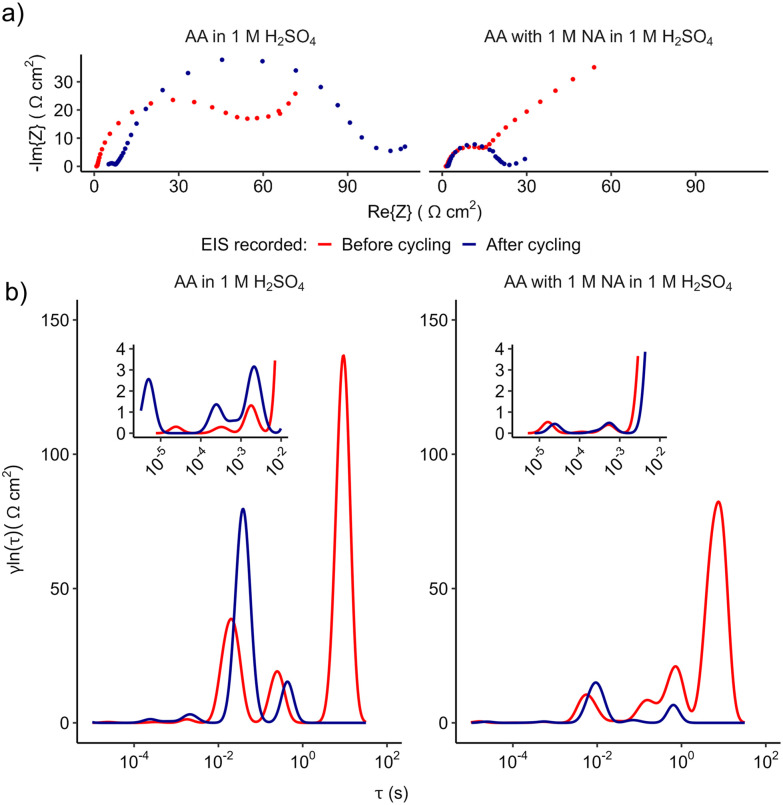
EIS of full- and symmetric-cells (a) −Im(*Z*) *vs.* Re(*Z*) for EIS of full cells before and after cycling. (b) *γ*ln *τ vs. τ* for the DRT transformed spectra of full cell EIS data before and after cycling. All EIS measurements recorded using PEIS with perturbation amplitude 10 mV. For full cell data, frequency range 200 kHz–0.1 Hz. The DRT transformation was performed using Gaussian Radial Basis Functions, with second-order regularization, all cells were run using carbon paper electrodes, the exact surface area of which cannot be known but is estimated to be 25 cm^2^.

While the addition of NA improved cycling stability for both AB and AA, the effect is particularly pronounced for AA. In order to understand the differences in cycling performance and the primary degradation mechanism for AA (with and without NA), electrochemical impedance spectroscopy (EIS) was used to explore the evolution of the electrochemical properties of interphases. EIS spectra were recorded before and after full-cell cycling (cell cycling conditions as previously described) both with and without 1 M NA. Fig. 6a shows the raw EIS data as Nyquist plots recorded before (red) and after (blue) cycling for full cells both with and without NA after 200 cycles. At least two semi-circles are observed in all the Nyquist plots, along with a feature at low frequencies, which appears either as the onset of a large semi-circle, or a straight line. For cells containing NA, the semi-circular region at high- to mid-frequencies appears stable and is present before and after cycling, while the low frequency regime changes significantly, moving from a Warburg-like straight line towards a curved feature of significantly lower magnitude. For cells without NA, the EIS response over the entire frequency range measured appears to be significantly altered by cycling, indicating major evolution of interphases at one or more parts of the cell.

Deconvolution of the EIS response of redox flow full cells is complicated significantly by the cell design and operating conditions. The flow cell provides an electrochemical system with many interphases, charge transfer and transport processes, as well as flow effects, which may contribute to the recorded EIS data.^39^ Here we apply two EIS analysis methods to aid deconvolution of the impedance contributions – Distribution of Relaxation Times (DRT) and Generalised Phase Element (GPE) analyses. DRT provides an overview of the number of polarisation relaxation processes involved in the EIS data and how each polarisation relaxation process varies before and after cycling.^40^Fig. 6b presents the full-cell time-domain DRT spectra, generated by transforming the frequency-domain EIS data. The DRT spectra show at least 6 peaks, indicating at least 6 charge polarisation relaxation mechanisms, making definite assignment of each peak difficult. However, comparison of these spectra for the electrolytes before (Fig. 6b, red) and after (Fig. 6b, blue) cycling allows some qualitative conclusions to be drawn.

The inserts in Fig. 6b show processes at faster time constants. These peaks (*τ* < 0.01 s) are typically assigned to either charge transport through an interphase or charge transfer.^40^ In cells without NA there is a large difference before (red) and after (blue) cycling, as all peaks grow significantly after cycling, showing an increased contribution to interphase impedance or charge transfer in the system. However, with NA there is almost no change before and after cycling, suggesting that the relaxation processes here are stable with respect to cycling. This indicates that the degradation processes have a reduced impact on charge transfer and interphase charge transport processes for cells containing NA, potentially suggesting that any film formed at an interphase during cycling (*e.g.*, on the membrane or electrode) is less dense and still allows permeation of electrolyte to the electrode and through the membrane for cells containing NA. This can also be explained as a reduction in the electrochemically active surface area due to formation of an electrically insulating, impermeable film on the electrode surface after cycling cells without NA, resulting in increased interphase and charge transfer impedance. Further discussion of the DRT results can be found in the SI.

**Fig. 7:**
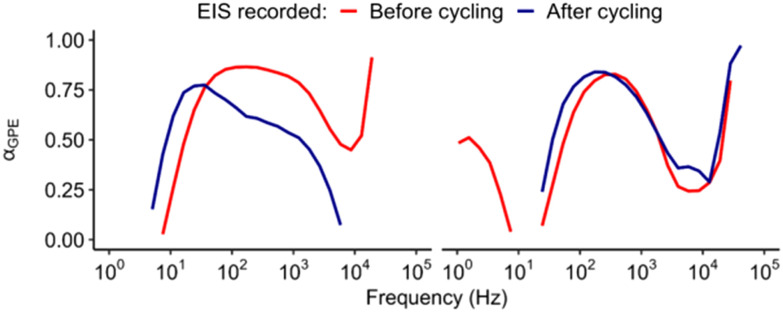
GPE analysis of EIS data before (red) and after (blue) cycling *α*_GPE_*vs.* Frequency, presents the frequency dependence of the *α*_GPE_ exponent, where a value of 1 equates to purely capacitive behaviour and a value of 0 equates to purely resistive behaviour.

Extracting information from the frequency-dependant capacitance using GPE models allows access to information regarding the nature of the observed fast *τ* peaks in the DRT.^41^ The slope of the imaginary component of the impedance can be related to the parameter *α*_GPE_, where this parameter can be interpreted as the ‘degree of capacitive/resistive’ behaviour shown by the electrochemical system at a particular frequency.^41^ Further information on the definition of *α*_GPE_ can be found in the Experimental Details and Methods. The frequency dependence of *α*_GPE_ can be seen in Fig. 7, a value of 1 equates to purely capacitive behaviour and a value of 0 equates to purely resistive behaviour. Before cycling (Fig. 7, red line) both systems with and without NA show a peak *α*_GPE_ value of 0.8 at *ca.* 100 Hz (without NA there is a more significant plateau), and a ‘dip’ down (at *ca*. 10 000 Hz) before trending towards 1 at higher frequencies. Without NA the system ‘dips’ to *ca.* 0.5, a value which can be interpreted as diffusion limited behaviour, suggesting a diffusive limitation to the high frequency charge transfer process for AA without NA. With NA this drop is more significant (to *ca.* 0.25), indicative of an increase in charge transfer compared to capacitive charge accumulation. After cycling, as frequencies increase, the *α*_GPE_ value in systems without NA approaches 0, indicating a lack of interphase charge-transfer processes due to the lack of any capacitive contribution, instead suggesting purely bulk resistive behaviour is seen. This result suggests that without NA, the interphase charge-transfer processes are blocked during cycling; this can be explained by considering deposition of an electrically insulating film at an electrochemically active interphase. With NA charge transfer is seen after cycling (there is comparatively little change to the frequency variation of *α*_GPE_ during cycling in the mid to high frequency range), showing that NA prevents or at least minimises this charge-transfer blocking deposition.^42^ Further work using GPE analysis in support of these conclusions can be found in the SI and Fig. S14.

**Fig. 8:**
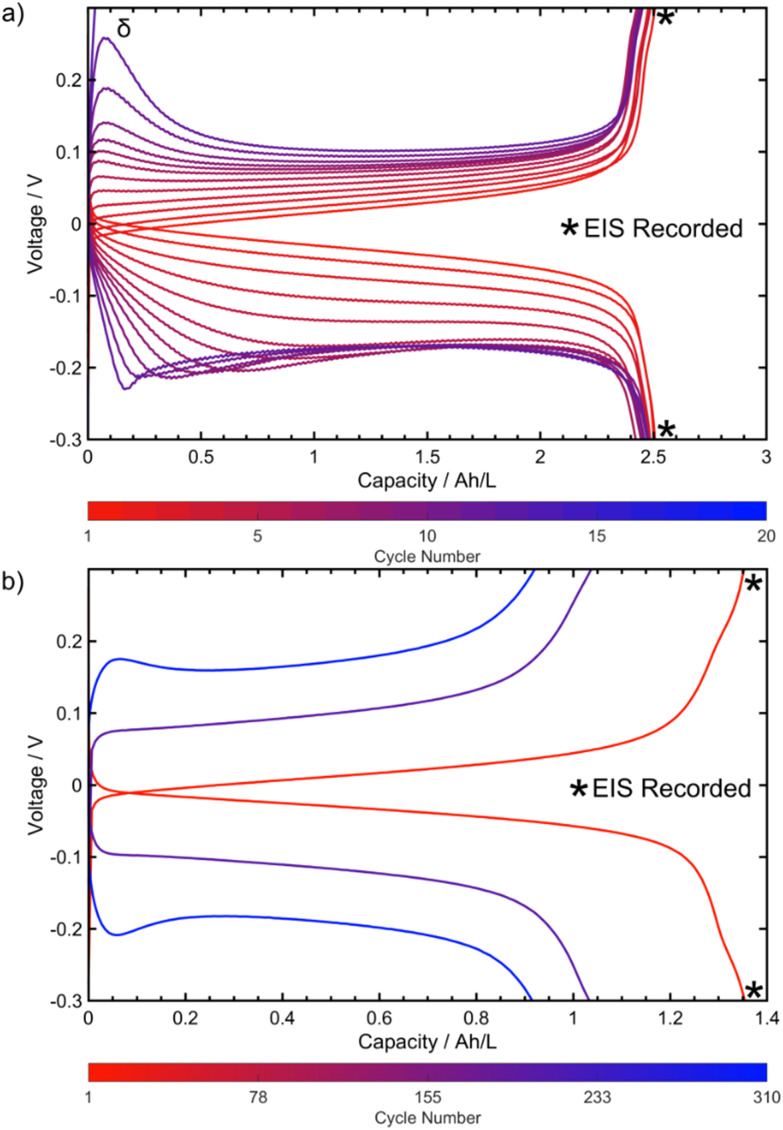
Symmetric cell cycling data, where the catholyte was initially discharged against 300 mM VCl_2_ and 75 mM VCl_3_. This catholyte was then cycled against a fully charged AA tank acting as the anolyte in a rebuilt cell for 100 mM AA in 1 M H_2_SO_4_ with (a) no NA and (b) 1 M NA, between −0.3 and 0.3 V with a current of 50 mA (10 mA cm^−1^). In (a), a feature, *δ*, is marked in the voltage *versus* capacity profile. EIS measurements were performed after cycling for the charged cell.^42^

To explore degradation mechanisms where vanadium crossover has been eliminated as a capacity-loss mechanism, symmetric cells were built using the AA^2+^ in both 1 M H_2_SO_4_ alone and 1 M H_2_SO_4_ with 1 M NA. Without NA in the supporting electrolyte the symmetric cell had a very short electrochemical lifetime. A peak at the start of the charge cycle (marked *δ* in Fig. 8a) grows in over the first 12 cycles, while overpotentials rapidly increased out of the voltage range of the cell. Impedance measurements taken after cycling show a massive increase in Ohmic resistance (Fig. S15). This behaviour can be explained by the deposition of a degradation layer at an interphase (either the membrane or the electrode), which eventually prevents charge-transfer processes from taking place. Conversely, when NA is present the symmetric cell is very stable (Fig. 8b). There is still some capacity loss, though less than seen in full-cell cycling, confirming that crossover also makes a contribution to capacity fade. This capacity loss could in part be due to adsorption of the positive AA+ ions on the negatively charged NA (spoiling of the membrane was observed after cycling), though this effect was not quantified, based on the minimal capacity loss observed in the symmetric cell, it is believed not to be a major contribution to overall capacity loss during cycling. The same peak growth (*δ*) can be seen only after an extended potential hold during charging and discharging in the presence of NA, which presumably accelerates the degradation. *In situ* EIS, measured at the top of charge and discharge, was also performed and DRT analysis of these results in support of these conclusions can be found in Fig. S16.

We propose that there is deposition, most likely of a polymeric film on the electrode/membrane during cycling, that accounts at least in part for the observed capacity fade. Capacity is lost both through the consumption of redox-active material and through the blocking of the membrane and electrode interfaces, preventing crucial charge transfer processes. Polymerisation of phenothiazines has been well documented in the literature, reportedly proceeding *via* a 1e^−^ oxidation to a radical cation (typically taking place at voltages greater than 1 V), which then forms intermolecular C–N and N–N bonds leading to the cross-linked oligomeric films.^43,44^ The initial 1e^−^ oxidation has sometimes been found to be reversible, depending on phenothiazine substituents and pH. For example, in methylene blue the same film-forming process is not as easily induced. Additionally, at higher pH the reaction has been found to be slower.^43,44^ Combining the insights from DRT and GPE analyses, it can be concluded that in cells both with and without NA there is some film formation (there is an increase in charge accumulation and therefore capacitance during EIS measurements). However, the presence of NA prevents a complete passivation of the electrode. The NA clearly supresses polymerisation of AA, most likely by disrupting the π stacking of AA and preventing extended aggregation, as is supported in this work by the NMR discussed previously. However, further discussion of the exact mechanism with which NA and AA interact is beyond the scope of this work.

## Conclusions

Phenothiazines represent an exciting group of compounds for catholytes in AORFBs, but thus far only one example, MB, has been reported in aqueous conditions. The electrochemical performance of three commercial dyes, TOB, AA and AB, as catholytes in AORFBs is reported here in 1 M H_2_SO_4_ with and without the hydrotrope additive nicotinamide (NA). It was found that NA not only improved solubility of the dyes but also improved electrochemical stability. Regardless of cycling conditions, AA did not, however, reach its theoretical capacity when concentrations of 100 mM of AA were used. Dimer formation was confirmed by spectroscopic studies, providing an explanation for the reduction in accessible capacity as only one ion per dimer can be fully oxidised/reduced under the voltage windows used here. The increased steric bulk of the fully methylated functional groups in MB prevent this dimerization, but also limit positive interaction with NA. Further studies are in progress to understand this dimerisation process in more detail.

Extensive studies were undertaken to understand the primary degradation mechanism and charge-limiting mechanisms in the AA and AA/NA system (the most promising of these candidates). This work applies DRT and is, to the best of our knowledge, the first to apply GPE EIS methods to AORFBs and has demonstrated the valuable role these techniques can have in understanding the degradation processes taking place at RFB electrodes and membrane interphases. Importantly, it may not be possible to observe these processes by only studying the electrolyte solution either *ex situ* or *in situ;* a combination of both must be used.

These results should be of general value to the applications of phenothiazine dyes containing amino functional groups in AORFBs. By understanding the degradation mechanism, it was possible to demonstrate the role of NA in preventing polymerisation. Understanding the role of amine substituents in the polymerisation and subsequent capacity loss in cells is key for molecular design when considering future work with phenothiazines in AORFBs. The phenothiazine most explored in this work, Azure-A (AA), is a non-toxic and cheap material which exhibits promisingly stable electrochemistry and high solubility, given appropriate supporting electrolyte conditions.

## Author contributions

N. L. F.: investigation, methodology, software, validation, visualisation, writing – original draft. K. M.: investigation, software, visualisation, writing – review & editing. D. H.: investigation, writing – review & editing. K. S.: investigation, validation, writing – review & editing. D. S. W.: conceptualisation, funding acquisition, supervision, writing – review & editing. C. P. G.: conceptualisation, funding acquisition, supervision, writing – review & editing.

## Conflicts of interest

There are no conflicts to declare.

## Supplementary Material

EB-002-D5EB00223K-s001

## Data Availability

Further data supporting this article has been included in the supplementary information (SI). Supplementary information is available. The supplementary information contains, (i) the Beer–Lambert Law, (ii) electrochemical data for all dyes tested (cyclic voltammetry and cell cycling), (iii) DFT predicted UV/vis spectra of Azure-A, and (iv) Electrochemical Impedance detail including generalised phase element analysis (GPE) and EIS of symmetric cells. See DOI: https://doi.org/10.1039/d5eb00223k. MATLAB code was used to process and plot some electrochemical data presented in this article, this can be found at https://github.com/Q-sci-chem/EC-lab-. The code used to process and plot remaining data (EIS) can be found at https://github.com/kmylrea/Redlox-flow-EIS-scripts.
