# Supplementary material for: The behaviour of phenothiazines as catholytes in aqueous-organic redox flow batteries
Source: EES Batter. 2026 Jun 17. Online ahead of print. doi: 10.1039/d5eb00223k (PMC13312259; doi:10.1039/d5eb00223k)
Supplement: EB-OLF-D5EB00223K-s001 [file EB-OLF-D5EB00223K-s001.pdf]

Supplementary Information (SI) for EES Batteries.
This journal is © The Royal Society of Chemistry 2026

Supporting Information:

The Behaviour of Phenothiazines as Catholytes in Aqueous-Organic Redox Flow Batteries

Nadia L. Farag[1], Kieran C. Mylrea[1], Dominic Hey[1], Kawarpal Singh[1], Dominic S. Wright[1], Clare P. Grey[1]

1- Yusuf Hamied Department of Chemistry University of Cambridge, Lensfield Rd, Cambridge, CB2 1EW (United Kingdom)

Contents

Supporting Information: 1
The Behaviour of Phenothiazines as Catholytes in Aqueous-Organic Redox Flow Batteries 1
1.1 Beer-Lambert Law 2
1.2 Initial Electrochemical Data 2
1.3 Spectroscopy 8
1.3.1 DFT Predicted UV/Vis spectra 12
1.4 Electrochemical Impedance 12
1.4.1 Generalised Phase Element Analysis (GPE) 12
1.4.2 Symmetric Cells 14

1.1 Beer-Lambert Law

The Beer-Lambert law is given in the following equation: A = εcl, where A is the measured absorbance (no units), ε the molar absorptivity ($M^{-1}cm^{-1}$), c the concentration (M) and l the path length (cm, 1 cm in this case).[34] Calibration curves of known concentrations were used to calculate ε, which can be taken to be the slope (an example for Azure B can be seen in Figure S1). A saturated solution of each dye was then prepared and diluted by a known amount (as the saturated solutions were too opaque to measure directly) before measuring the absorption, using the obtained value for ε a concentration of the saturated solution was obtained.

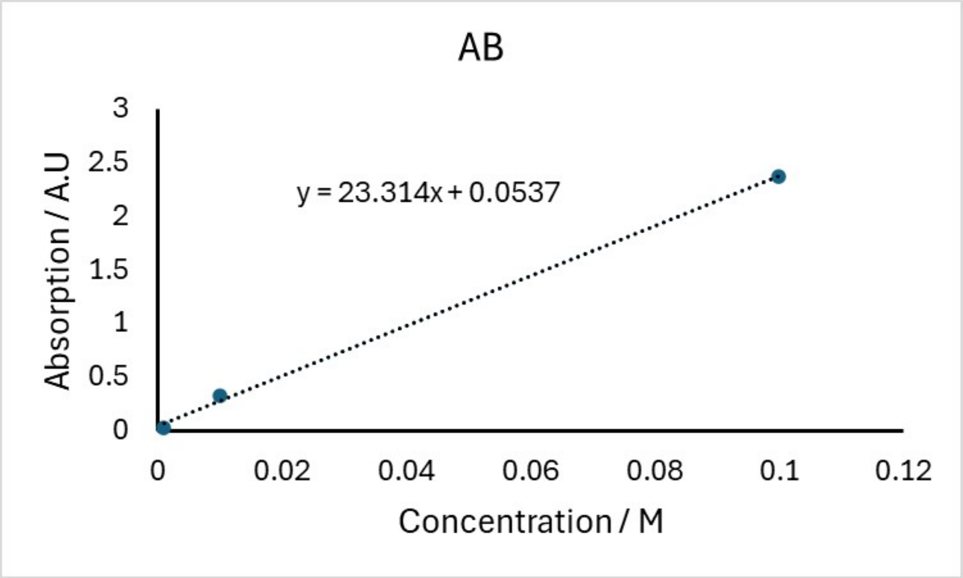

Figure S 1: Example of calibration curve obtained for Azure-B (AB) using 0.001, 0.01 and 0.1 M AB in 1 M H_2SO_4, the subsequent trendline and equation can be seen, all absorption values are averaged over three measurements.

1.2 Initial Electrochemical Data

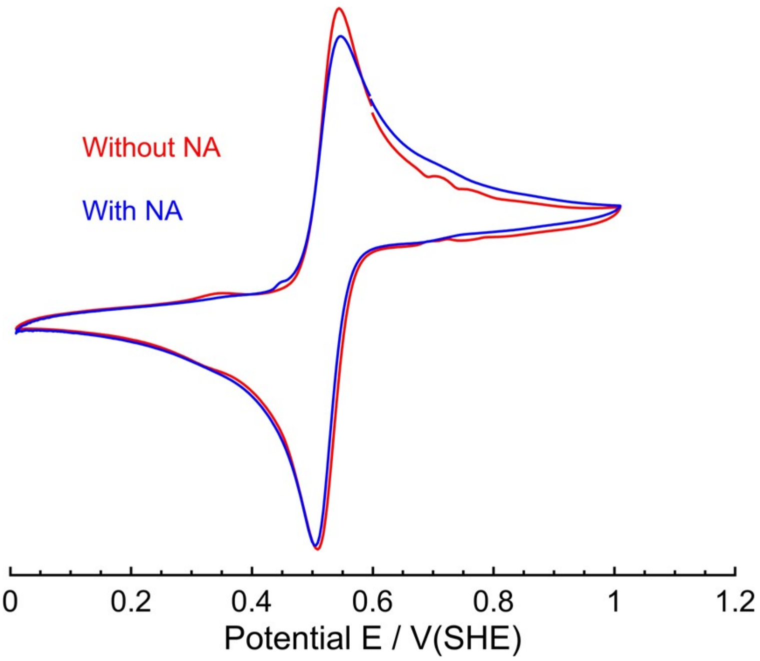

Figure S 2: CV of 1 mM AA in 1 M H_2SO_4 without NA (red trace) and with 1 M NA (blue trace).

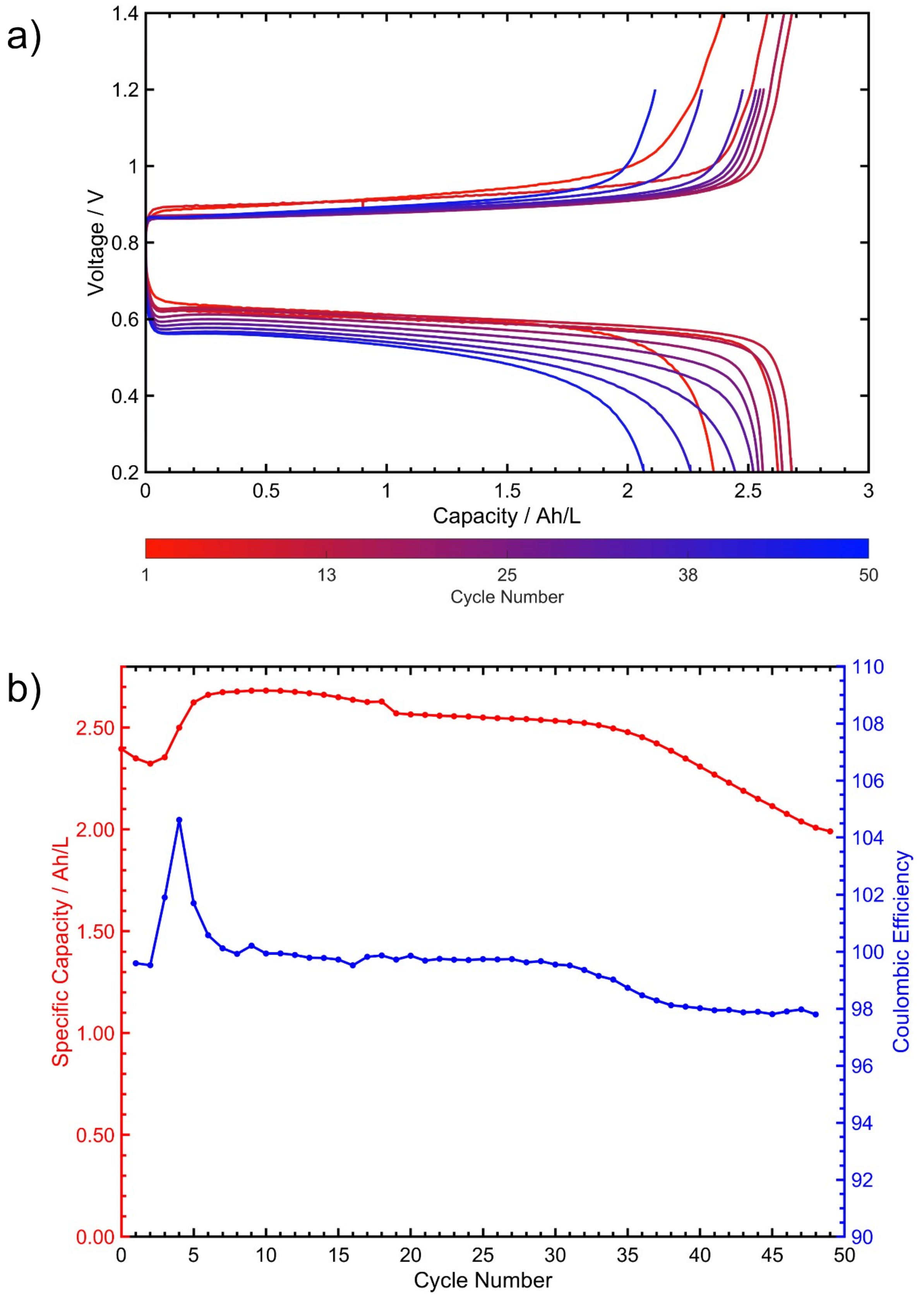

Figure S 3: a) Cycling of 100 mM AA in 1 M H_2SO_4 with 1 M NA, two sheets of Nafion 212 membrane, b) Capacity (red) and coulombic efficiency (blue).

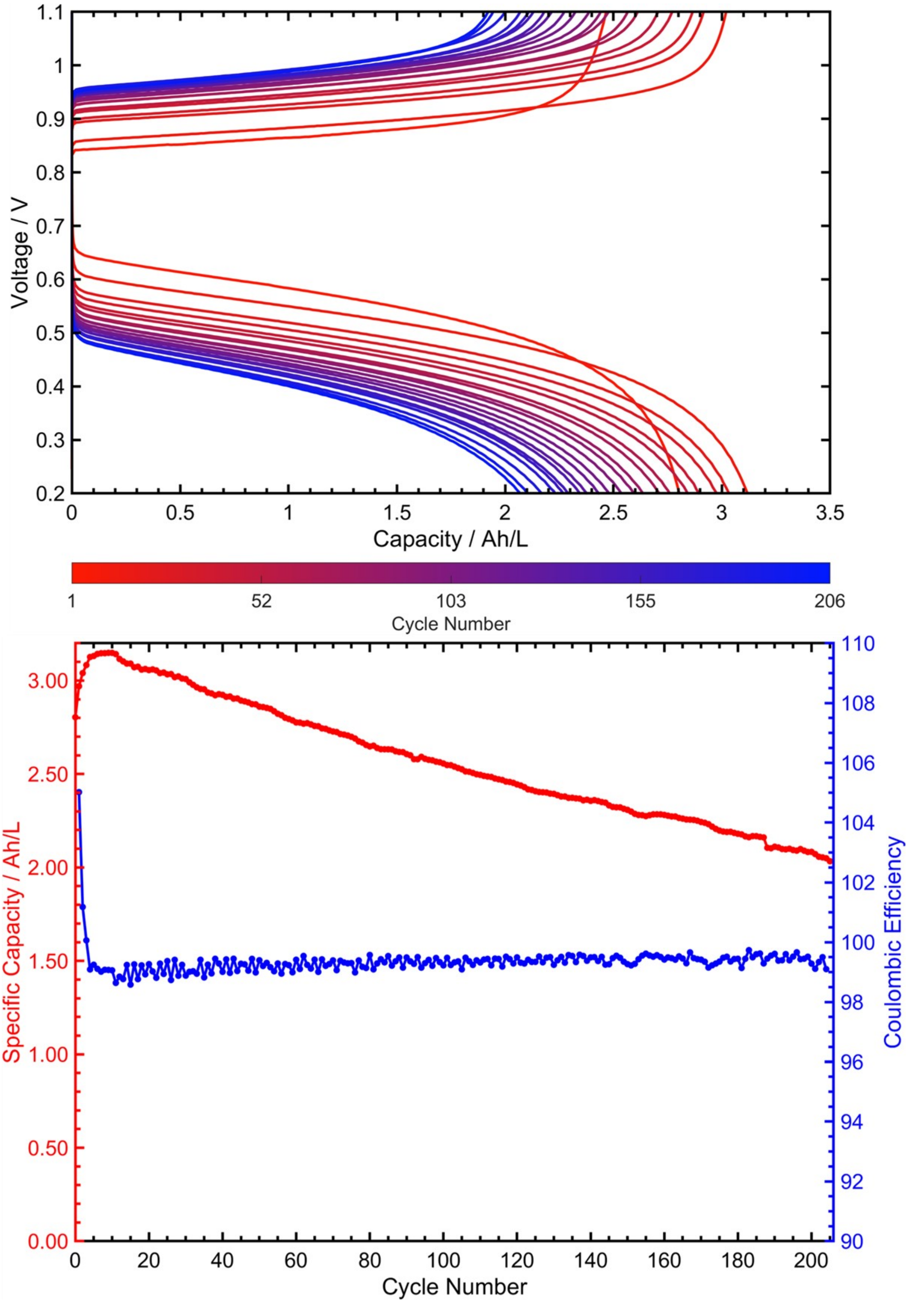

Figure S 4: a) Cycling of 100 mM TOB in 1 M H_2SO_4 with 1 M NA, one sheet of Nafion 212 membrane, b) Capacity (red) and coulombic efficiency (blue).

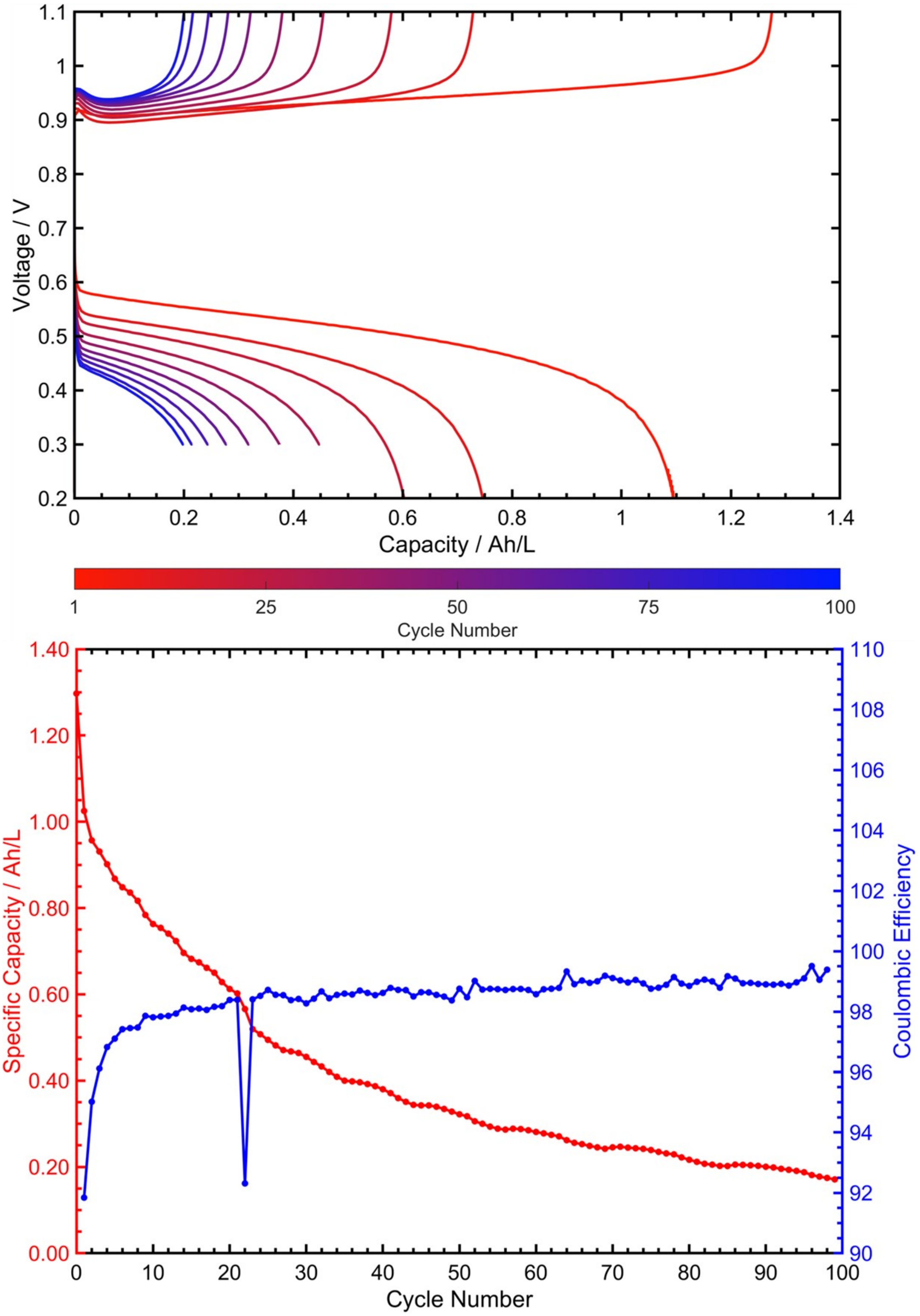

Figure S 5: a) Cycling of 100 mM AB in 1 M H_2SO_4, one sheet of Nafion 212 membrane, b) Capacity (red) and coulombic efficiency (blue).

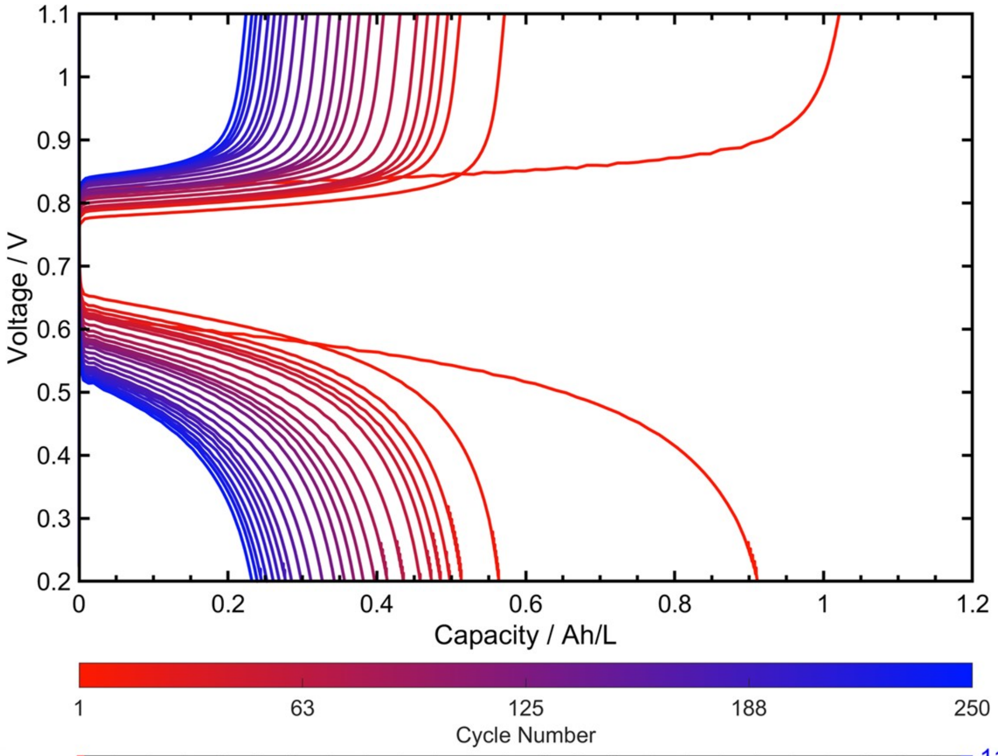

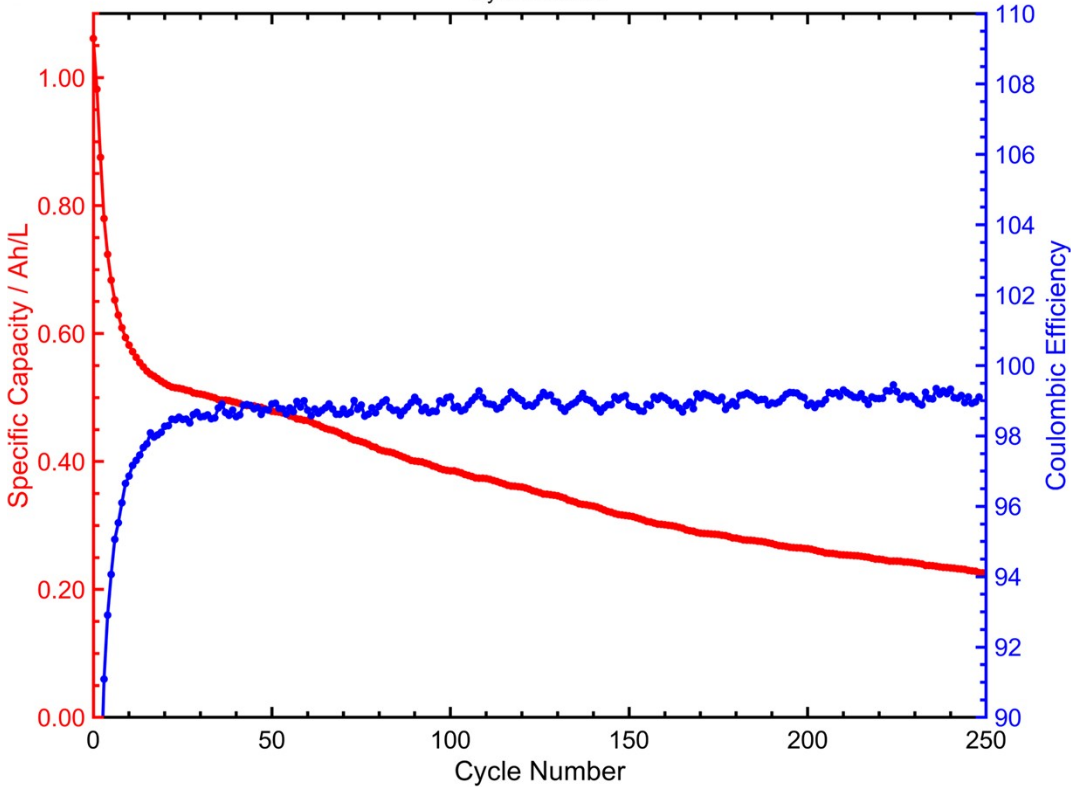

Figure S 6: a) Cycling of 100 mM AB in 1 M H_2SO_4 with 1 M NA, one sheet of Nafion 212 membrane, b) Capacity (red) and coulombic efficiency (blue).

1.3 Spectroscopy

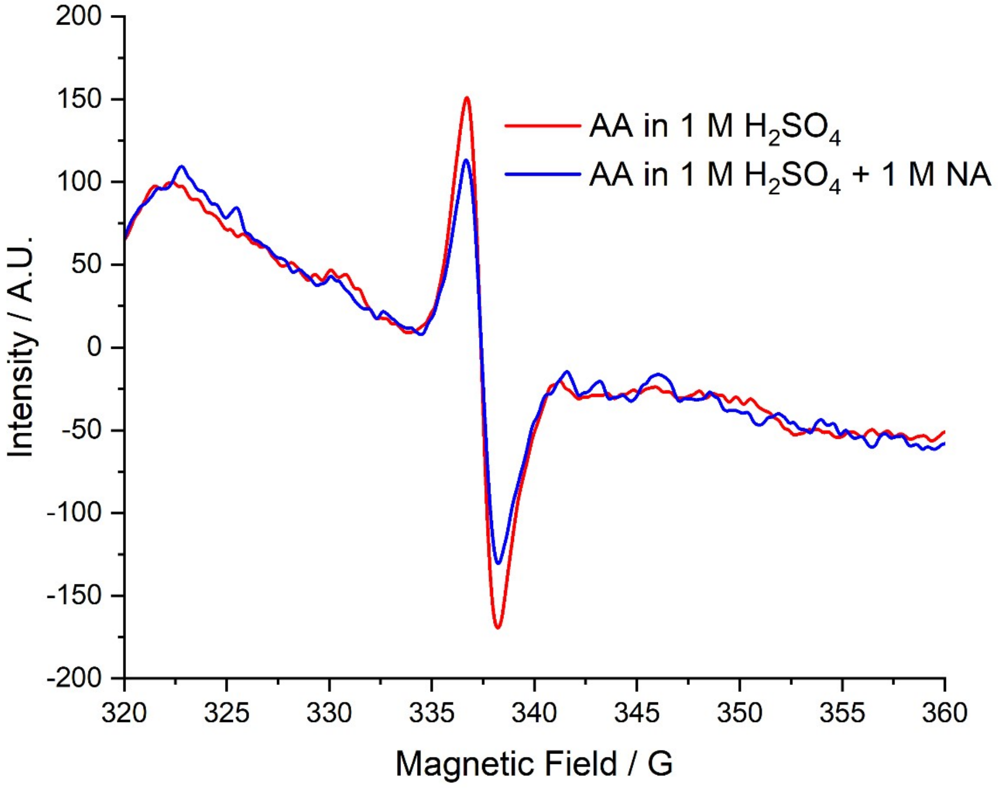

Figure S 7: EPR trace of AA in 1 M H_2SO_4 (red) and AA in 1 M H_2SO_4 + 1M NA.

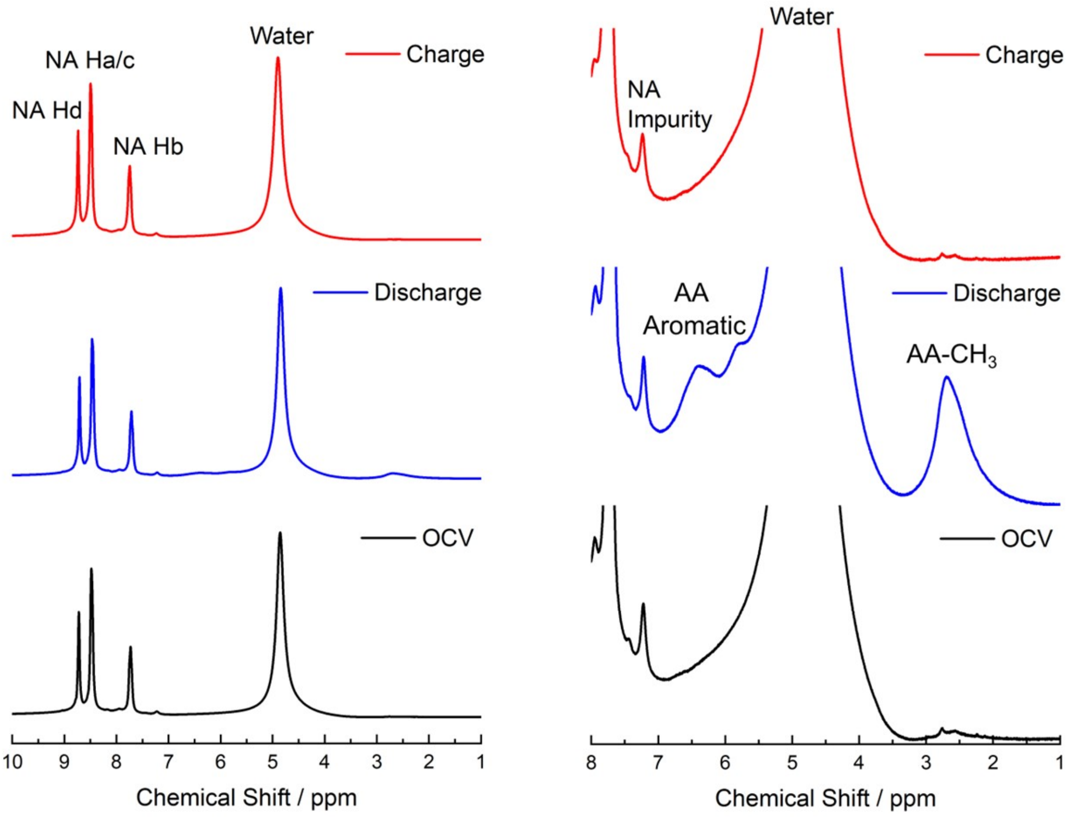

Figure S 8: Slices from the in situ ^{1}H NMR spectra (shown in Figure 4.3b of the main text) showing the spectra at OCV (black), the end of discharge (blue) and the top of charge (red). The ^{1}H resonances from NA are labelled on the right; weaker peaks are seen in the zoom shown on the right which are assigned to H_2AA^{2+} and an impurity in NA. Arrows indicate the extremely broad and weak resonances due to AA^{2+}. Some of the resonances are obscured by the intense and broad water resonance.

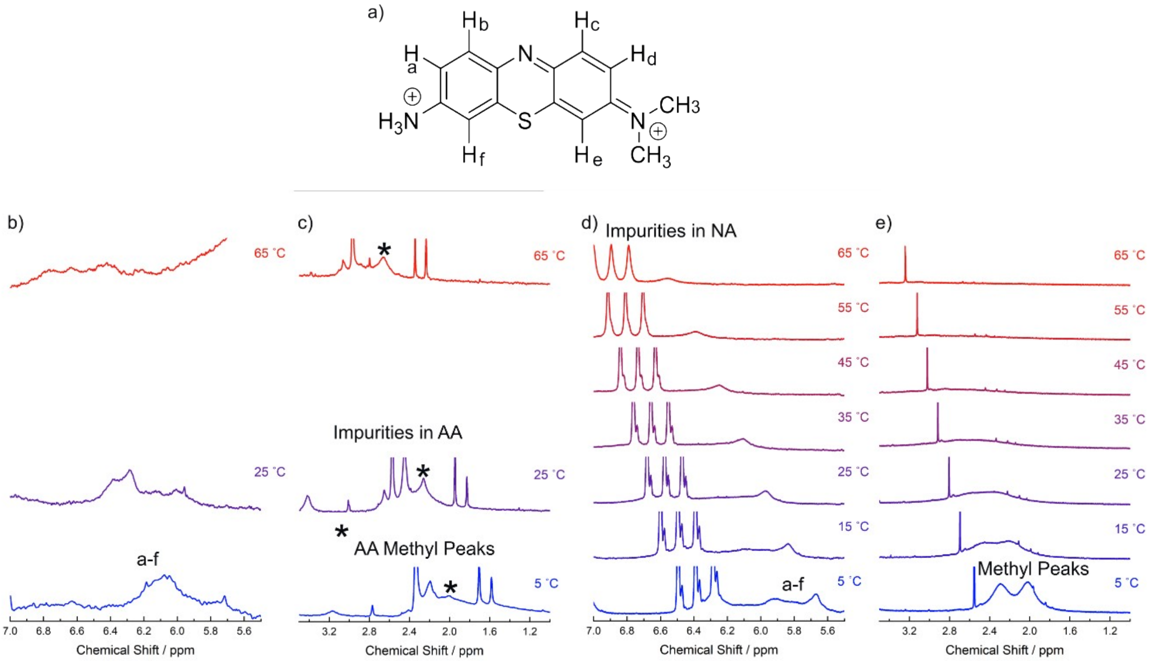

Figure S 9: a) Labelled molecular diagram of AA in the fully oxidised state (AA^{2+}) as it would be in 1 M H_2SO_4, Variable-temperature ^1H NMR in D_2O with 1 M H_2SO_4 between 5 and 65°C for b) aromatic AA peaks, c) methyl AA peaks, d) aromatic AA peaks with 1 M NA in solution, e) methyl AA peaks with 1 M NA in solution.

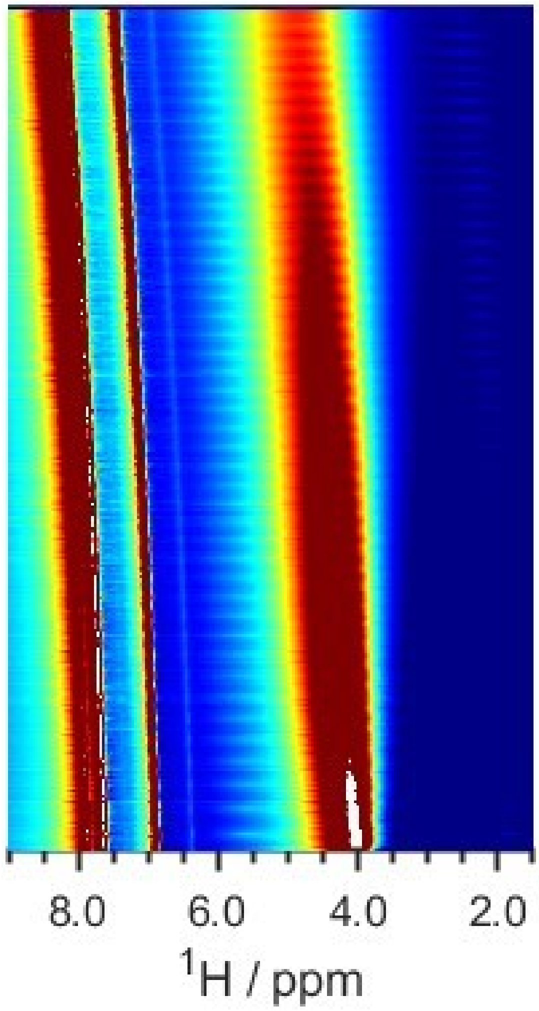

Figure S 10: In-situ ^1H NMR data over ca. 50 charge/discharge cycles for 100 mM AA in 1 M H_2SO_4 with 1 M NA against VCl_2, showing the broadening of the water peak (at ca. 4 ppm) caused by vanadium crossover and the build-up of the V^{4+} radical.

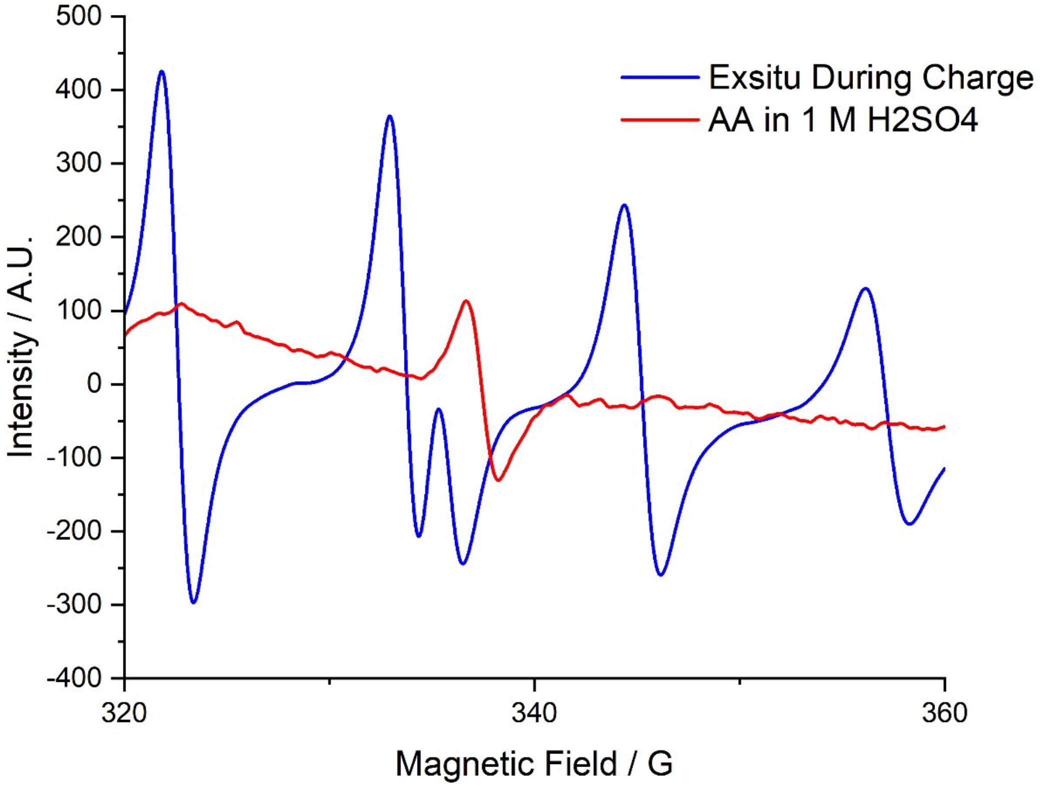

Figure S 11: EPR trace of AA in 1 M H2SO4 (red) and ex situ spectra of 200 mM AA in 1 M H2SO4 with 1 M NA in H_2O, taken during charge (blue).

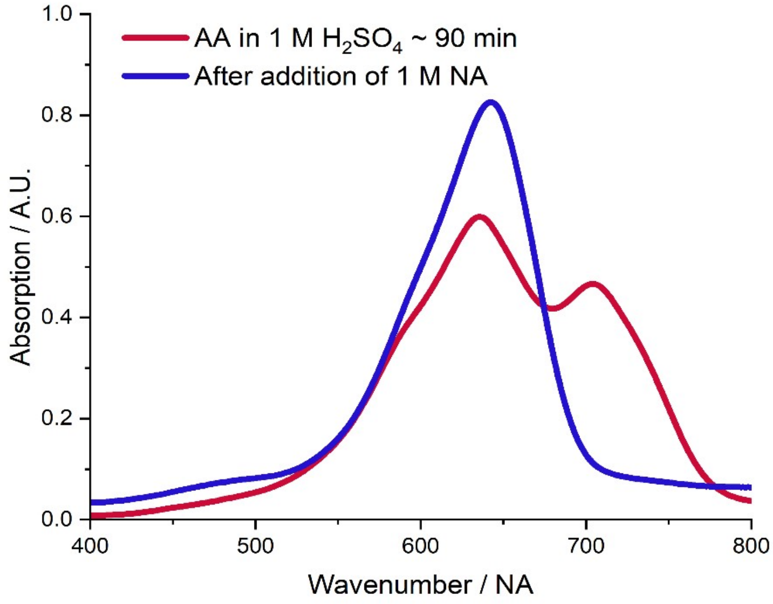

Figure S 12: UV/vis of 0.01 mM AA in 1 M H_2SO_4 without NA (red trace) and with 1 M NA (blue trace).

1.3.1 DFT Predicted UV/Vis spectra

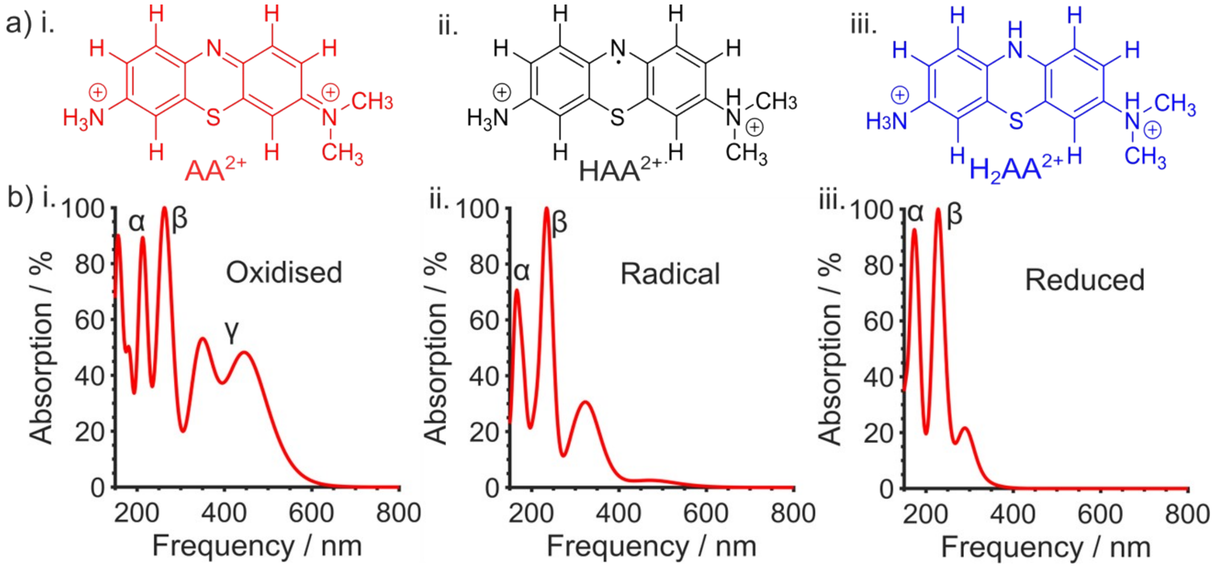

Figure S 13: a) AA in the i. fully-oxidised (charged) state (AA^{2+}), ii. radical state ($HAA^{2+\cdot}$) and iii. fully-reduced (discharged) state (H_2AA^{2+}), b) DFT predicted UV vis spectra for AA in the i. fully-oxidised (charged) state (AA^{2+}), ii. radical state ($HAA^{2+\cdot}$) and iii. fully-reduced (discharged) state (H_2AA^{2+}). The three characteristic groups of peaks are observed in the experimental spectra, α, β and γ.

1.4 Electrochemical Impedance

1.4.1 Generalised Phase Element Analysis (GPE)

Specifically, the further information on the resistive/capacitive nature of interphases can extracted by calculation of the frequency-dependant capacitances from the raw EIS data. GPE analysis relies on the derivation of models for the EIS response of different morphologies (of dispersive-time-constant behaviour) at an interphase; utilising different models involves comparing different numbers of dimensions of heterogeneity at an interphase.[41] A “2D” model for capacitance extracted by GPE can represent a 2D surface with heterogeneous charge transfer and transport properties, for example, a surface with different grains or defects showing differing charge transport properties. A “3D” model of capacitance extracted from GPE analysis represents an interphase which is heterogeneous in three dimensions, such as a porous interphase or an interphase with different chemistries present throughout the interphase. Further details on GPE analysis can be found in the Experimental Details and Methods.

Figure S 14 shows the calculated frequency-dependant capacitance, with the dashed and solid lines indicating 2D and 3D time-constant dispersion, respectively. Before cycling both systems with and without NA have an excellent match between 2D and 3D time constant distributions,

indicating that there is a 2D distribution of time constants contributing to the EIS response in this region. Both systems with and without NA exhibit a slope which plateaus - the onset of this slop can reveal kinetic information as higher onset frequency indicates faster charge transfer kinetics. In this case the slope in systems without NA develops *ca.* 10 Hz, in NA this occurs *ca.* 30 Hz, suggesting faster charge transfer kinetics with NA. In further support of this conclusion, without NA the plateau slopes slightly upwards as frequency increases, suggesting increased kinetic limitation for charge transfer kinetics at higher frequencies.

After cycling in both cases, a 3D distribution trends sharply upwards at higher frequencies, this suggests that the charge transfer process observed pre-cycling at these frequencies has been at least partially blocked. With NA this feature is also captured by a 2D distribution of time constants, indicating minimal 3D contribution. Without NA this feature is not captured at all by a 2D distribution, strongly suggesting there is a thicker or denser deposit at the interphase causing the blocking of high frequency charge transfer - whereas with NA, the lack of additional 3D heterogeneity suggests that the 2D charge transfer heterogeneity observed pre-cycling is still dominant.

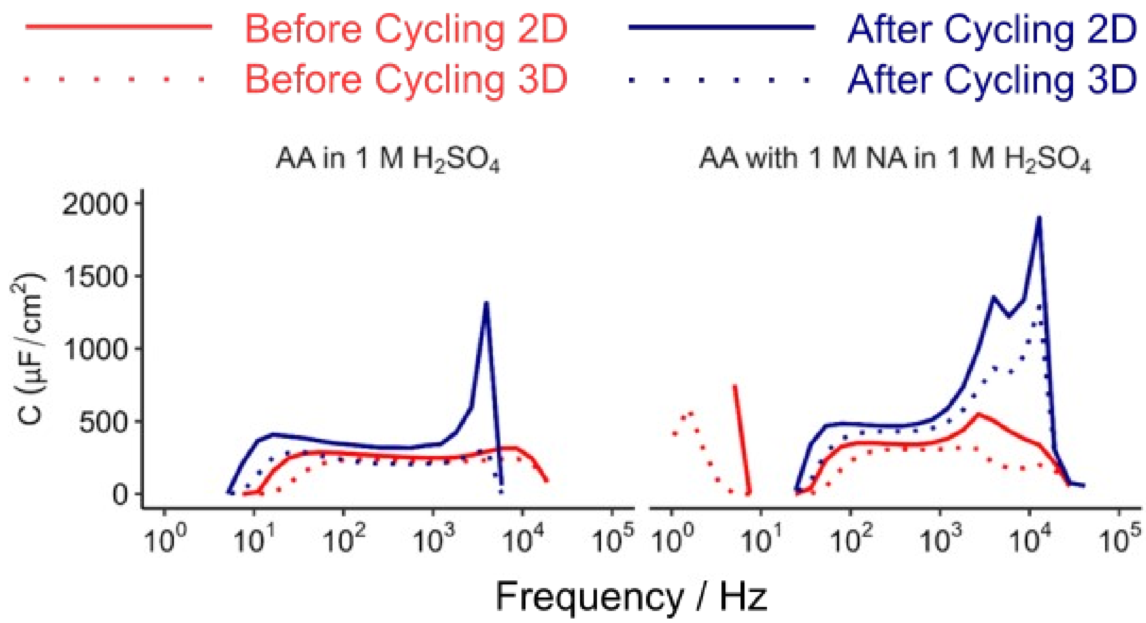

Figure S 14: Capacitance vs Frequency comparing frequency-dependant capacitance calculated using a 2D and 3D distribution of interphase time constants for full cell EIS data before and after cycling.

1.4.2 Symmetric Cells

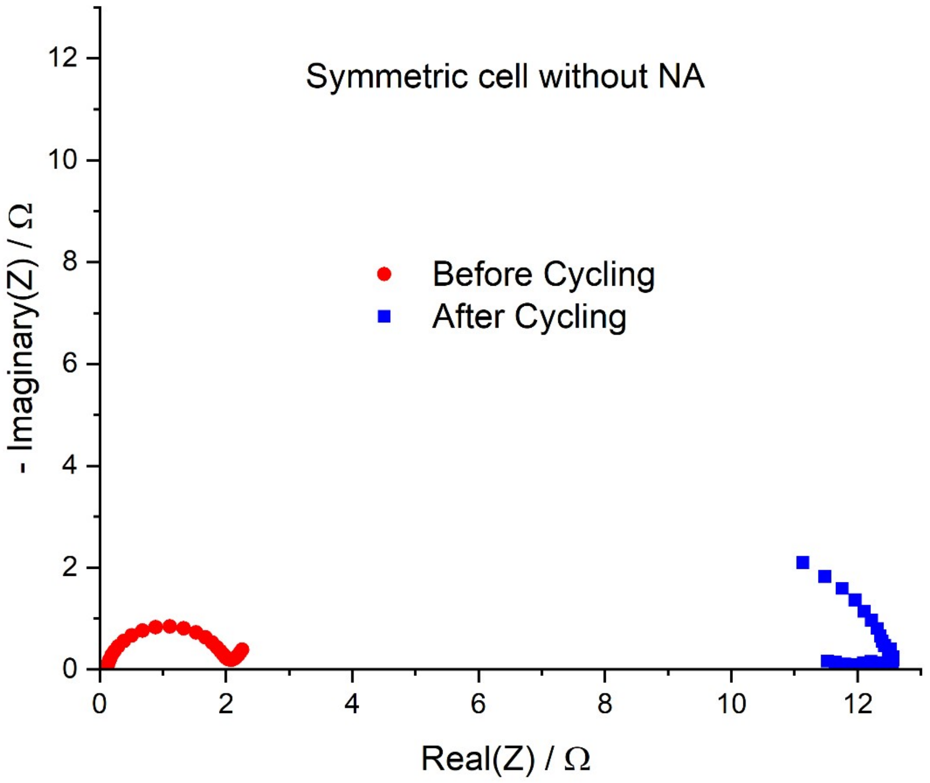

Figure S 15: -Im(Z) vs Re(Z) for EIS of Symmetric cell without NA before (red) and after (blue) cycling.

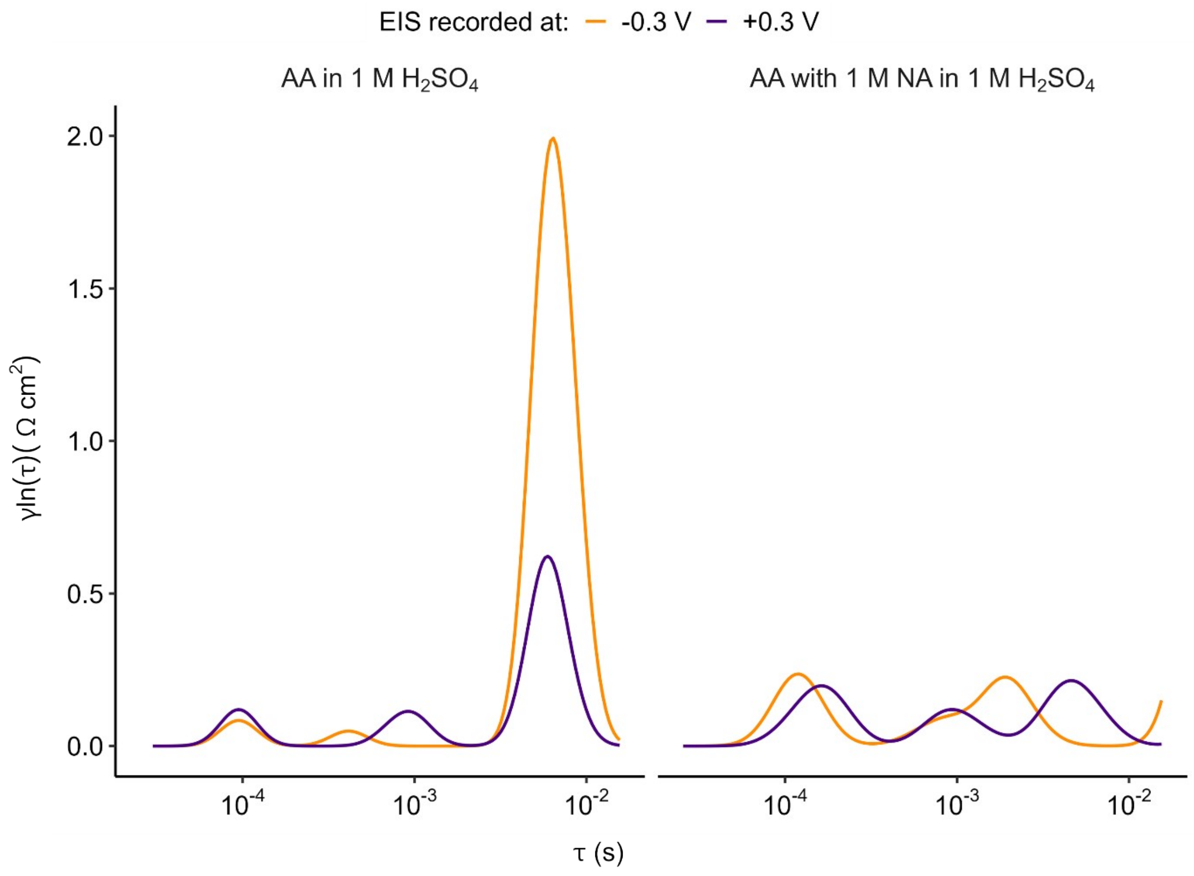

Figure S 16: DRT-transformed EIS data of the symmetric cells reveal peaks at similar t values to those seen in full cell measurements (Figure 6b). The symmetric cells also reveal significantly less variation in DRT peak intensity between the two voltages for cells containing NA. This may suggest faster kinetics in NA- containing cells, allowing the charge polarisation relaxation processes responsible for these peaks to be in equilibrium at both voltages at the onset of the EIS measurement. We speculate that this is not the case for cells without NA, resulting in significant change in intensity, in particular for the peak at t ca. 0.01 s. The peaks with t < 0.01 s are typically assigned to either charge transport through an interphase or charge transfer.1
